# Risk factors for hospital-acquired pneumonia in hip fracture patients: a systematic review and meta-analysis

**DOI:** 10.1186/s12891-023-07123-0

**Published:** 2024-01-02

**Authors:** Wei Yao, Xiaojia Sun, Wanyun Tang, Wei Wang, Qiaomei Lv, Wenbo Ding

**Affiliations:** 1 Department of Orthopedics, Dandong Central Hospital, China Medical University, No. 338 Jinshan Street, Zhenxing District, Dandong, Liaoning Province 118002 People’s Republic of China; 2 Department of Oncology, Dandong Central Hospital, China Medical University, Dandong, China; 3 Department of Pediatrics, Dandong Central Hospital, China Medical University, Dandong, China

**Keywords:** Hip fracture, Hospital-acquired pneumonia, Risk factors, Meta-analysis

## Abstract

**Objective:**

This study aimed to systematically assess the incidence and risk factors for hospital-acquired pneumonia (HAP) in hip fracture patients by meta-analysis.

**Methods:**

Systematically searched four English databases (PubMed, EMBASE, The Cochrane Library, and Web Of Science) and four Chinese databases (CNKI, CQVIP, Sinomed, and WAN FANG) from inception until 20 November 2023. All studies involving risk factors of HAP in patients with hip fractures were considered. Newcastle-Ottawa Scale was used to evaluate the quality of the included studies. The results were presented with the pooled odds ratio (OR) and 95% confidence interval (95% CI).

**Results:**

Of 35 articles (337,818 patients) included in this study, the incidence of HAP was 89 per 1000 cases. Twenty-three risk factors were eventually involved in the meta-analysis, and 21 risk factors were significant. Our study has identified four significant risk factors (advanced age, preoperative time, COPD, and hypoalbuminemia) associated with HAP, as follows: Advanced age as a continuous variable (OR 1.07, 95% CI 1.05–1.10), Advanced age > 70 years (OR 2.34, 95% CI 1.77–3.09), Advanced age > 80 years (OR 2.98, 95% CI 2.06–4.31), Chronic obstructive pulmonary disease (COPD) (OR 3.44, 95% CI 2.83–4.19), Time from injury to operation as a continuous variable (OR 1.09, 95% CI 1.07–1.12), Time from injury to operation ≥48 h (OR 3.59, 95% CI 2.88–4.48), Hypoalbuminemia < 3.0 g/dL (OR 3.03, 95% CI 1.93–4.73), and Hypoalbuminemia < 3.5 g/dL (OR 2.68, 95% CI 2.15–3.36). However, it is important to note that all the studies included in our research were retrospective in nature, which introduces certain limitations to the level of evidence and the ability to establish causal inferences.

**Discussion:**

Patients who have suffered hip fractures are at an increased risk of developing postoperative hospital-acquired pneumonia, which can lead to prolonged hospital stays and adverse clinical outcomes. Consequently, the identification of these risk factors offers novel insights and methodologies for healthcare professionals in terms of both prevention and treatment.

**Trial registration:**

Registration number: INPLASY2022100091.

**Supplementary Information:**

The online version contains supplementary material available at 10.1186/s12891-023-07123-0.

## Introduction

Hip fractures are a major public health concern, with approximately 4.5 million cases worldwide annually and an expected increase to 21 million by 2060 [1]. Hip fractures are associated with a high mortality rate, reaching 8.4–36% within 1 year of the fracture over the age of 70 [2]. Complications during hospitalization, including hospital-acquired pneumonia (HAP), can further increase the risk of mortality in these patients [3–5]. HAP is defined as pneumonia that occurs 48 hours or more after admission to the hospital, and it is one of the most common and essential complications in hip fracture patients [6–8]. Epidemiological evidence shows that the incidence of postoperative HAP after hip fracture typically ranges from 5 to 15%, and that HAP in hip fracture patients increases mortality by 27–43%, length of hospital stay by 56%, and the risk of readmission by 8-fold [6, 9]. Furthermore, there is limited research on strategies for preventing HAP. These strategies may include early mobilization after surgery, oral care, inhalation prophylaxis measures, and the use of prophylactic antibiotics. The implementation of clinical preventive strategies is hindered by the presence of ambiguous underlying risk factors. Therefore, identifying the risk factors for HAP in hip fracture patients and preventing its occurrence is essential for optimizing perioperative care, predicting postoperative outcomes, and reducing mortality [10].

Previous studies and meta-analyses have explored potential risk factors for pneumonia in hip fracture patients after hospitalization. However, the limitations of these studies include small sample sizes (anemia, ASA ≥ III) and a lack of inclusion of Chinese literature (gender, age, anemia, ASA ≥ III, duration of surgery, length of hospital stay, and some laboratory biomarkers [2, 11–14]), which may restrict the generalizability of the research findings and increase the risk of selection bias and geographical bias. Moreover, many studies only provide a summary of the risk factors for HAP following hip fracture, without conducting a more comprehensive investigation into these risk factors (such as subgroup analysis). This heterogeneity in previous study design may mislead the conclusions. To address this issue and improve comparability, the present study conducted subgroup analyses for risk factors with high heterogeneity.

This meta-analysis aims to investigate and summarize the risk factors for HAP in hip fracture patients by including more literature and employing rigorous statistical methods. It report all risk factors currently associated with HAP and further explore important risk factors to help clinicians identify high-risk patients for early and targeted treatment to prevent HAP.

This study aims to examine two primary queries: (1) What is the incidence of hospital-acquired pneumonia among individuals with hip fractures? (2) Which risk factors are associated with the development of hospital-acquired pneumonia in patients with hip fractures?

## Methods

This study was conducted under the Preferred Reporting Items for Systematic Reviews and Meta-Analyses (PRISMA) statement [15].

### Search strategy

Systematically Searched four English databases (PubMed, EMBASE, The Cochrane Library, and Web Of Science) and four Chinese databases (CNKI, CQVIP, Sinomed, and WAN FANG) from inception until 20 November 2023. All studies involving risk factors of HAP in patients with hip fractures were considered using a search strategy that combines keywords and free words. To avoid omitting the literature, we have reduced the restrictions on medical subject words and added more free words. The main medical subject words were as follows: “Hip”, “Hip Fractures”, “Femoral Neck Fractures”, and “Pneumonia”. Simultaneously, the references of included studies and relevant reviews were manually reviewed.

### Eligibility criteria

Inclusion criteria were as follows: (1) Study types: Cohort study or case-control studies; (2) Participants: All the patients with hip fractures who have been hospitalized; (3) Outcomes: Original studies that explore the relationship between demographic factors, comorbidity factors, surgical factors, and laboratory factors with hospital-acquired pneumonia; (4) Data: Full text can be obtained, and sufficient data were published for estimating an odds ratio (OR) with 95% confidence interval (95% CI) by multivariate logistic regression.

Exclusion criteria were as follows: (1) Study types: Those studies that are reviews, letters, comments, case reports, abstracts, and animal trials; (2) Participants: Patients with hip fractures were hospitalized for less than 48 hours or caused by polytrauma; (3) Outcomes: The risk factors associated with HAP were unreported; (4) Data: Duplicate data or unable to calculate odds ratio (OR) with 95% confidence interval (95% CI).

### Data extraction

After removing duplicate records from the retrieved literature, the titles and abstracts of all articles were independently reviewed by the researchers based on risk factors. Upon meeting the inclusion criteria, the full texts underwent further evaluation. If the full-text screening was also successful, the researchers extracted the following data (factors identified through multivariate logistic regression): first author’s name, year of publication, country, study type, number of cases, number of patients with HAP, incidence of HAP, mean age of patients and controls, male-to-female ratio of patients and controls, as well as significant risk factors. Additionally, odds ratios (ORs) and 95% confidence intervals (CIs) were extracted. Two researchers (WY and XJS) independently conducted the entire process, quantifying inter-reviewer agreement using the Kappa coefficient to ensure unbiased evaluation. Any discrepancies were resolved through thorough discussion to reach a consensus. If consensus couldn’t be reached, an independent arbitrator (WBD) was consulted for resolution.

### Quality assessment

**Fig. 1:**
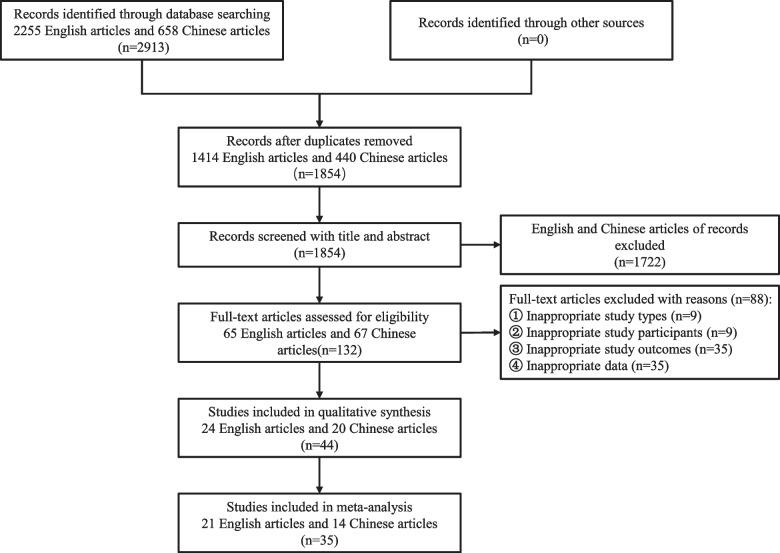
Flow diagram of studies screening

The Newcastle–Ottawa scale (NOS) was used to evaluate the quality of the included study, mainly based on three items: the selection of the study population (0–4 stars), the comparability between groups (0–2 stars), and the measurement of exposure outcomes (0–3 stars). The overall score of NOS is between 0-9stars, and ≥ 6 stars were considered a high-quality study. Two researchers (WY, XJS) were independently assess the included studies’ quality. Finally, 35 articles (including 21 English articles and 14 Chinese articles) with research quality ≥6 stars were included in the meta-analysis (inter-reviewer agreement abstracts kappa = 0.82 ± 0.03; full-texts kappa = 0.66 ± 0.05) (Fig. 1). The disagreements between the two authors were resolved by discussion with the third author (QML).

### Statistical analysis

We excluded risk factors that were only reported in a single publication. Subsequently, two authors (WY and XJS) decided to group together identical or nearly identical risk factors. The adjusted OR with a 95% CI from the original studies was extracted by both authors and recorded in a standardized data extraction table. Statistical analyses were conducted to examine the effect estimates of both the adjusted and unadjusted studies, with the aim of determining if any significant differences existed. In cases where only frequency data were provided, the ORs and CIs were independently calculated by the two authors. Any articles with missing relevant data were addressed by contacting the corresponding authors; otherwise, they were excluded. Disagreements were settled through discussions and negotiations between the two authors. If unresolved, consultations were held with the senior researcher (WBD).

The consistency index (I^2^) was used to evaluate the statistical heterogeneity between studies. When I^2^ < 50% or Q-test *P* > 0.1, the fixed effect model was used; When I^2^ > 50% or Q-test *P* < 0.1, a random effect model was used, indicating heterogeneity between studies. The effect of individual studies that yield meta-analysis estimates by omitting one study at a time to characterize the extent to which removing individual studies affects the estimates (Sensitivity analysis). Subgroup analyses were employed to ascertain the relationship between postoperative HAP after hip fracture and related study characteristics (Advanced age, Hypoalbuminemia and the number of comorbidities) as a possible source of heterogeneity. When ten or more studies were included, the publication bias was evaluated by funnel plots and Begg’s and Egger’s tests. *P* < 0.05, and asymmetric funnel plots indicated significant publication bias. *P* value < 0.05 in the overall effect test suggests that the risk factors were statistically significant.

Review Manager version 5.3 (The Cochrane Collaboration, Oxford, UK), STATA 15.0 (STATA Corporation, College Station, TX, USA), and R software version 4.0.3 (R 4.0.3 for Windows; GitHub, San Francisco, USA) were used for all statistical analyses.

## Results

### Study characteristics and quality assessment

The essential characteristics of the included studies are shown in Table 1. A total of 35 articles have been included since 2015, including 25 case-control studies and 10 cohort studies. The included articles comprised retrospective studies, and subgroup analysis did not reveal any significant heterogeneity. The study population was drawn from 6 countries, with the majority being from Asian countries (China and Korea), while 7 articles originated from Europe and the United States. Notably, the articles from Asia primarily focused on advanced age and COPD, whereas the articles from Europe and the US primarily examined sex and the time from injury to operation. The summary of risk factors of hospital-acquired pneumonia (HAP) reported in these studies is shown in Table 2. A total of 43 risk factors were reported, with advanced age mentioned in 19 articles, and time from injury to operation, COPD, and hypoalbuminemia mentioned in 14 or more articles.

**Table 1:** The basic characteristic of the included studies

Study	Country	Study type	Sample size			Mean age (Years)		Gender (Male/Female)		Significant factors	NOS score
			Total	HAP No.	HAP (%)	HAP	NHAP	HAP	NHAP		
Byun et al. 2018	Korea	case–control study	432	38	8.80	83.70 ± 7.8	78.60 ± 8.2	13/25	112/282	Advanced age, High BMI, Hypoalbuminemia, Duration of surgery, Time from injury to operation	8
Shin et al. [2]	Korea	cohort study	1155	59	5.11	83.08 ± 7.3	77.90 ± 10.05	21/38	295/801	Advanced age, CVA, Hypoalbuminemia	8
Ahn et al. [39]	Korea	case–control study	1208	47	3.89	79.70 ± 8.2	79.20 ± 7.5	19/28	294/867	Postoperative delirium, ASA, Charlson Comorbidity Index, Male sex, Hypoalbuminemia	8
Bohl et al. 2017	USA	cohort study	29,377	1191	4.05	NA	NA	NA	NA	Advanced age, Male sex, High BMI, CVA, COPD, Dyspnea on exertion, Functional status, Anemia	8
Wilson et al. [13]	USA	cohort study	5673	64	1.13	NA	NA	NA	NA	Hypoalbuminemia	7
Danford et al. [35]	USA	cohort study	27,058	893	3.30	NA	NA	282/611	7958/18207	Time from injury to operation	7
Ekström et al. [14]	Sweden	cohort study	1915	144	7.52	NA	NA	65/79	415/1356	Male sex, COPD, Cognitive function dysfunction	9
Meyer et al. [56]	Sweden	cohort study	170,193	9049	5.32	NA	NA	NA	NA	ASA	9
Salarbaks et al. [9]	Netherland	cohort study	407	62	15.23	84.00 ± 7.9	83.00 ± 6.7	33/29	247/98	Male sex, COPD	8
Glassou et al. [36]	Denmark	cohort study	72,520	3805	5.25	NA	NA	NA	NA	Time from injury to operation	8
Chang et al. [17]	China	case–control study	240	25	10.42	NA	NA	9/16	68/147	Advanced age, History of stroke, History of cancer, Platelet, Hyperglycemia	8
Deng et al. [18]	China	case–control study	9806	1977	20.16	NA	NA	919/1058	3008/4821	Advanced age, Number of comorbidities, Male sex	9
Wang et al. [40]	China	case–control study	293	33	11.26	84.50 ± 3.2	85.10 ± 3.4	20/13	76/184	Male sex, Hypoalbuminemia, Low oxygen level	9
Xiang et al. [37]	China	case–control study	1113	166	14.92	86.40 ± 5.8	78.80 ± 7.2	53/113	331/616	High BMI, High c-reactive protein, Functional status, Time from injury to operation	8
Zhang et al. [19]	China	case–control study	758	82	10.82	NA	NA	27/55	223/453	Advanced age, COPD, Type of anesthesia	8
Zhang et al. [31]	China	case–control study	1285	70	5.45	82.00 ± 5.8	79.00 ± 6.7	30/40	359/856	COPD, Number of comorbidities, ASA, Functional status, Cognitive function dysfunction	9
Chen et al. [20]	China	case–control study	1008	87	8.63	NA	NA	32/55	277/644	Advanced age, Time from injury to operation, History of smoking, ASA, COPD, Hypoalbuminemia, High RDW, Time of Mechanical ventilation, ICU	9
Ding et al. 2019	China	case–control study	2251	61	2.71	NA	NA	34/27	891/1299	Hypoalbuminemia, NISS, Postoperative bed rest time	8
Liu et al. [21]	China	case–control study	230	23	10.00	NA	NA	13/10	107/100	Advanced age, Duration of surgery, Type of anesthesia, Time from injury to operation	7
Jiang et al. [22]	China	case–control study	545	28	5.14	82.80 ± 6.7	79.00 ± 7.5	12/16	150/367	Advanced age, High BMI, History of stroke, Duration of surgery, Time from injury to operation, Hypoalbuminemia	8
Ying et al. 2015	China	case–control study	1419	72	5.07	82.00 ± 9.6	76.00 ± 9.6	23/49	522/825	Advanced age, Male sex, ASA, Type of anesthesia, Anemia, Hypoalbuminemia, High Cr, COPD, History of cancer	8
Wang et al. [24]	China	case–control study	720	54	7.50	NA	NA	24/30	212/454	Advanced age, COPD, History of cancer, History of stroke, Time from injury to operation	7
Wei et al. [25]	China	case–control study	392	56	14.29	82.30 ± 7.1	78.20 ± 7.0	24/32	113/223	Advanced age, COPD, Time from injury to operation, Type of operation, Type of anesthesia	7
Wei et al. [26]	China	case–control study	469	48	10.24	NA	NA	19/29	149/272	Advanced age, Time from injury to operation, Type of anesthesia	7
Zhang et al. [27]	China	case–control study	224	20	8.93	NA	NA	14/6	137/67	Advanced age, Time from injury to operation, History of stroke, Duration of surgery, Type of anesthesia, History of smoking, Anemia	9
Zhu et al. [28]	China	case–control study	576	145	25.17	82.46 ± 5.56	79.22 ± 6.51	45/100	98/333	Advanced age, Hypoalbuminemia, CVA	7
Yuan et al. [32]	China	case–control study	207	43	20.77	81.30 ± 7.2	79.70 ± 7.7	16/27	44/120	Anemia, History of stroke, COPD	7
Zhu et al. [38]	China	case–control study	741	26	3.51	NA	NA	20/6	314/401	ASA, Time from injury to operation	8
Lv et al. [11]	China	cohort study	1429	70	4.90	82.00 ± 9.6	74.00 ± 11.9	22/48	575/784	Advanced age, Type of fractures, Number of comorbidities, ASA, Type of operation, Hypoalbuminemia, High Cr, Mechanical ventilation	9
Wang et al. [24]	China	case–control study	720	54	7.50	82.30 ± 8.1	77.50 ± 8.5	20/34	273/393	Hypoalbuminemia, History of stroke, COPD, Time from injury to operation	9
Zhao et al. [4]	China	case–control study	1495	53	3.55	NA	NA	28/25	483/959	Advanced age, Male sex, COPD, Liver disease, Urinary tract infection, High CKMB, High d-dimer, High BNP	7
Ji et al. [34]	China	cohort study	901	55	6.10	81.60 ± 7.7	78.50 ± 7.0	23/32	280/566	COPD, History of stroke, Hypoxemia, Time from injury to operation	9
Lv et al. [29]	China	case–control study	526	56	10.65	75.81 ± 9.03	67.03 ± 7.11	17/39	151/319	Advanced age, History of smoking, Time from injury to operation, COPD, Hypoalbuminemia, High RDW, ICU, Time of Mechanical ventilation	8
Yu et al. [30]	China	case–control study	267	35	13.11	74.15 ± 10.23	65.08 ± 9.15	20/15	133/99	Advanced age, Hyperglycemia, Anemia, Hypoalbuminemia, Type of anesthesia, Duration of surgery	9
Zhang et al. [31]	China	case–control study	265	53	20.00	81.00 ± 8.9	81.00 ± 8.9	14/39	57/155	ICU, High RDW	9

*RV GLS* Right ventricular global longitudinal strain; *BMI* Body mass index; *CVA* Cardiovascular Accident; *ASA* American Society of Anesthesiologists status scale; *COPD* Chronic obstructive pulmonary disease; *RDW* Red blood cell volume distribution width; *ICU* Intensive care unit; *NISS* National institute of health stroke scale; *Cr* Creatinine; *CKMB* Creatine kinase MB blood; *BNP* B-natriuretic peptide; *NA* not available

**Table 2:** Detailed data on potential risk factors for hospital-acquired pneumonia

Potential risk	No. of studies	Included in meta-analysis	Included in heterogeneity	Included in sensitivity analysis	Included in subgroup analysis	Included in publication bias
Advanced age	19	•	•	•	•	•
Time from injury to operation	15	•	•	•	◦	◦
Hypoalbuminemia	14	•	•	•	•	•
COPD	14	•	•	•	◦	•
Male sex	8	•	•	•	◦	◦
ASA	7	•	•	◦	◦	◦
History of stroke	7	•	•	◦	◦	◦
Type of anesthesia	7	•	•	•	◦	◦
Anemia	5	•	•	•	◦	◦
Duration of surgery	5	•	•	•	◦	◦
High BMI	4	•	•	•	◦	◦
High RDW	3	•	•	◦	◦	◦
CVA	3	•	•	•	◦	◦
Functional status	3	•	•	•	◦	◦
History of cancer	3	•	•	◦	◦	◦
History of smoking	3	•	•	◦	◦	◦
ICU	3	•	•	◦	◦	◦
Number of comorbidities	3	•	•	◦	•	◦
Cognitive function dysfunction	2	•	•	◦	◦	◦
High Cr	2	•	•	◦	◦	◦
Hyperglycemia	2	•	•	◦	◦	◦
Time of Mechanical ventilation	2	•	•	◦	◦	◦
Type of operation	2	•	•	◦	◦	◦
Charlson Comorbidity Index	1	◦	◦	◦	◦	◦
Dyspnea on exertion	1	◦	◦	◦	◦	◦
High BNP	1	◦	◦	◦	◦	◦
High CKMB	1	◦	◦	◦	◦	◦
High c-reactive protein	1	◦	◦	◦	◦	◦
High d-dimer	1	◦	◦	◦	◦	◦
Hospital stay	1	◦	◦	◦	◦	◦
Hypoxemia	1	◦	◦	◦	◦	◦
Liver disease	1	◦	◦	◦	◦	◦
Low oxygen level	1	◦	◦	◦	◦	◦
Mechanical ventilation	1	◦	◦	◦	◦	◦
Nasoenteral tube	1	◦	◦	◦	◦	◦
NISS	1	◦	◦	◦	◦	◦
Platelet	1	◦	◦	◦	◦	◦
Postoperative bed rest time	1	◦	◦	◦	◦	◦
Postoperative delirium	1	◦	◦	◦	◦	◦
Preoperative modified frailty index	1	◦	◦	◦	◦	◦
RV GLS	1	◦	◦	◦	◦	◦
Type of fractures	1	◦	◦	◦	◦	◦
Urinary tract infection	1	◦	◦	◦	◦	◦

•: Included ◦: Excluded

*RV GLS* Right ventricular global longitudinal strain: *BMI* Body mass index: *CVA* Cardiovascular Accident: *ASA* American Society of Anesthesiologists status scale: *COPD* Chronic obstructive pulmonary disease: *RDW* Red blood cell volume distribution width: *ICU* Intensive care unit: *NISS* National institute of health stroke scale: *Cr* Creatinine: *CKMB* Creatine kinase MB blood: *BNP* B-natriuretic peptide

The methodological quality assessment included in the studies is shown in Table 1, using the NOS scale, with a score range of 0–9 stars. The quality assessment results of 35 studies were as follows: 9 stars in 12, 8 stars in 14, and 7 stars in 9. As a result, the quality of each study is higher. Detailed quality assessment results can be found in Supplementary eTable 1.

### Meta-analysis results

**Fig. 2:**
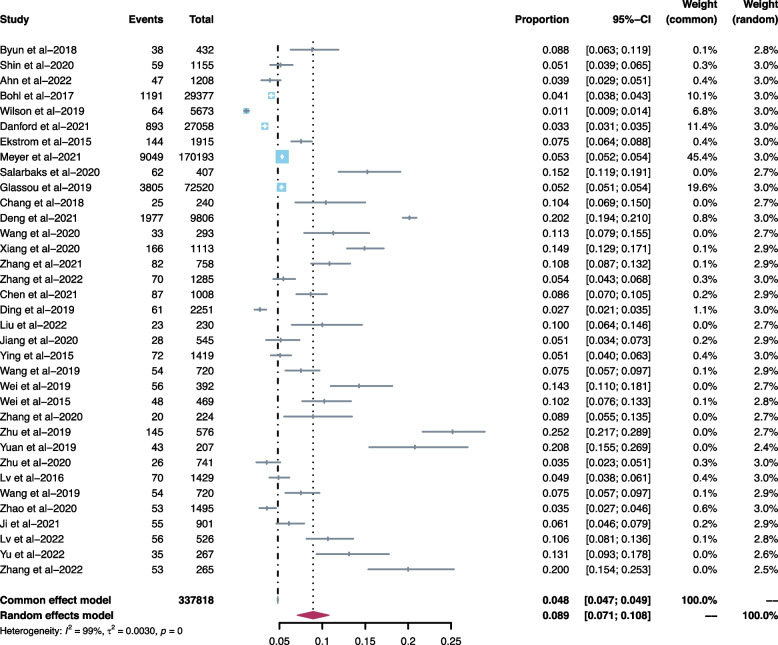
Forest plot for HAP incidence in 35 studies

The point incidence rate of HAP in 35 studies was between 1.1 and 25.2%, the overall cumulative incidence rate was 8.9% (95% CI: 0.071–0.108; I^2^ = 99%), and heterogeneity could not be solved by sensitivity analysis (Fig. 2). For the same risk factor, because the definition of each original study was different, some studies defined it as a continuous variable, while others defined it as a dichotomous variable. Therefore, we labeled the variable types of risk factors and combined the statistics respectively. When necessary, we also carried out a subgroup analysis for the same risk factors at different stratification levels (such as Advanced age and Hypoalbuminemia). Secondly, we divided the risk factors into four categories. In each category, there was a risk factor reported more than ten times by previous studies: Advanced age, COPD, Time from injury to operation, and Hypoalbuminemia. The detailed results of each factor are shown in Table 3.

**Table 3:** The Results of the meta-analysis of potential risk factors

Potential risk	variable	I^2^ (%)	Q-test (P)	Pooled OR	95% CI	*P*-value	Statistical Method
Demographics							
Advanced age	Continuous	22	0.27	1.07	1.05–1.10	< 0.001	Fixed
	Dichotomous	0	0.64	2.55	2.04–3.19	< 0.001	Fixed
Advanced age > 70 years	Dichotomous	0	0.70	2.34	1.77–3.09	< 0.001	Fixed
Advanced age > 80 years	Dichotomous	0	0.45	2.98	2.06–4.31	< 0.001	Fixed
60–69 years vs 70–79 years	Stratification	0	0.65	1.38	1.20–1.59	< 0.001	Fixed
60–69 years vs 80–89 years		10	0.29	1.82	1.59–2.09	< 0.001	
60–69 years vs ≥90 years		50	0.16	2.08	1.74–2.49	< 0.001	
Male sex	Dichotomous	0	0.76	2.04	1.78–2.34	< 0.001	Fixed
High BMI	Continuous	36	0.21	0.85	0.79–0.90	< 0.001	Fixed
Functional status	Dichotomous	0	0.76	3.13	2.11–4.63	< 0.001	Fixed
History of smoking	Dichotomous	0	0.58	2.89	2.34–3.57	< 0.001	Fixed
Comorbidity							
COPD	Dichotomous	17	0.27	3.44	2.83–4.19	< 0.001	Fixed
History of stroke	Dichotomous	0	0.95	3.10	2.28–4.20	< 0.001	Fixed
CVA	Dichotomous	0	0.35	1.56	1.26–1.93	< 0.001	Fixed
History of cancer	Dichotomous	0	0.56	3.77	2.13–6.67	< 0.001	Fixed
Number of comorbidities	Dichotomous	92	< 0.001	5.16	3.16–8.42	< 0.001	Random
Number of comorbidities = 1	Stratification	0	0.96	3.09	2.77–3.45	< 0.001	Fixed
Number of comorbidities = 2		0	0.40	7.42	6.24–8.84	< 0.001	
Number of comorbidities = 3		0	0.41	6.60	4.48–9.72	< 0.001	
Cognitive function dysfunction	Dichotomous	5	0.31	2.75	1.86–4.07	< 0.001	Fixed
Surgical							
Time from injury to operation	Continuous	2	0.36	1.09	1.07–1.12	< 0.001	Fixed
	Dichotomous	15	0.31	3.59	2.88–4.48	< 0.001	Fixed
ASA	Dichotomous	0	0.45	2.72	2.27–3.26	< 0.001	Fixed
Type of anesthesia	Dichotomous	16	0.31	0.24	0.18–0.32	< 0.001	Fixed
Duration of surgery	Continuous	89	0.003	1.02	1.00–1.03	0.01	Random
	Dichotomous	0	0.64	3.56	1.84–6.87	< 0.001	Fixed
ICU	Dichotomous	7	0.34	2.92	1.93–4.41	< 0.001	Fixed
Time of Mechanical ventilation	Continuous	71	0.06	4.48	1.89–10.64	< 0.001	Random
Type of operation	Dichotomous	0	0.74	5.03	2.58–9.81	< 0.001	Fixed
Laboratory							
Hypoalbuminemia	Dichotomous	29	0.16	2.75	2.25–3.36	< 0.001	Fixed
Hypoalbuminemia< 3.0 g/dL	Dichotomous	0	0.66	3.03	1.93–4.73	< 0.001	Fixed
Hypoalbuminemia< 3.5 g/dL	Dichotomous	45	0.07	2.68	2.15–3.36	< 0.001	Fixed
Anemia	Dichotomous	0	0.80	2.97	2.14–4.11	< 0.001	Fixed
High RDW	Continuous	0	0.43	3.14	2.35–4.20	< 0.001	Fixed
High Cr	Continuous	0	0.87	3.11	1.57–6.19	< 0.001	Fixed
Hyperglycemia	Dichotomous	86	0.007	6.39	0.58–70.06	0.13	Random

*OR* odds ratio; *CI* confidence interval; *BMI* Body mass index; *CVA* Cardiovascular Accident; *ASA* American Society of Anesthesiologists status scale; *COPD* Chronic obstructive pulmonary disease; *RDW* Red blood cell volume distribution width; *ICU* Intensive care unit; *Cr* Creatinine

### Patient factors - advanced age

**Fig. 3:**
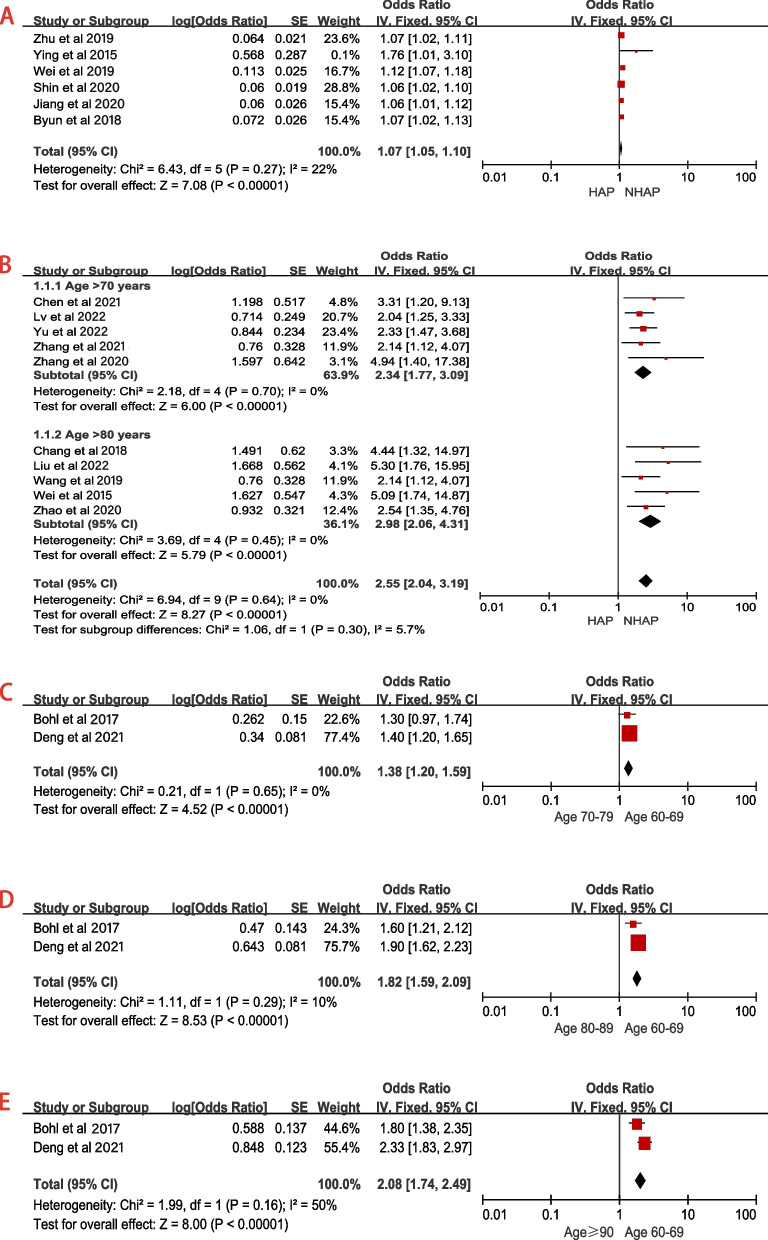
Forest plots for advanced age. **a** Sensitivity analysis for advanced age as a continuous variable (per year increase); **b** Subgroup analysis for advanced age as a dichotomous variable (age > 70 vs. ≤70 and age > 80 vs. ≤80); **c** Forest plot for advanced age as a stratification variable (60–69 years vs. 70–79 years); **d** Forest plot for advanced age as a stratification variable (60–69 years vs. 80–89 years); **e** Forest plot for advanced age as a stratification variable (60–69 years vs. ≥90 years)

Nineteen studies [2, 4, 6, 11, 16–30] reported the relationship between advanced age and HAP (Table 2), of which seven studies [2, 6, 11, 22, 23, 25, 28] reported the association between advanced age (continuous variable) and HAP. The results showed moderate heterogeneity among studies (*P* = 0.07, I^2^ = 49%; in Supplementary eFigure1. A). Sensitivity analysis was used to explore the source of heterogeneity. After deleting one of the articles (Lv et al. 2016 [11]), the heterogeneity between the studies decreased significantly (*P* = 0.27, I^2^ = 22%; Fig. 3A and Table 3). Summarizing the results of these studies showed that advanced age (continuous variable) was a risk factor for HAP in patients with hip fracture (Fixed-effects model; OR 1.07, 95% CI 1.05–1.10; Fig. 3A and Table 3).

Ten studies [4, 17, 19–21, 24, 26, 27, 29, 30] reported the relationship between advanced age (dichotomous variable) and HAP, of which five studies [19, 20, 27, 29, 30] reported the association between Age > 70 years and HAP, and the other five [4, 17, 21, 24, 26] reported the association between Age > 80 years and HAP. Subgroup analysis was conducted for the ten studies due to different levels among the studies. The results showed no heterogeneity between the studies (*P* = 0.64, I^2^ = 0%; Fig. 3B and Table 3). Further analysis showed that the incidence of HAP in patients over 80 years old was higher than that in patients over 70 years old (Age > 80: OR = 2.98 vs. Age > 70: OR = 2.34; Fig. 3B and Table 3). A funnel plot for advanced age (dichotomous variable) was used to evaluate publication bias (Fig. 5A). Since the visual method could not determine whether the funnel plot is symmetrical, we performed Begg’s and Egger’s tests (in Supplementary eFigure1.B&C) for advanced age (dichotomous variable). The results showed *P* > 0.05, indicating no publication bias among each subgroup.

The remaining two studies [6, 18] reported the relationship between advanced age (stratification variable) and HAP, and there was minor heterogeneity among the studies (Fig. 3C-E and Table 3). Compared with other age groups, patients older than 90 had an increased HAP risk (Fixed-effects model; OR 2.08, 95% CI 1.74–2.49; Fig. 3E and Table 3).

### Patient factors - COPD

**Fig. 4:**
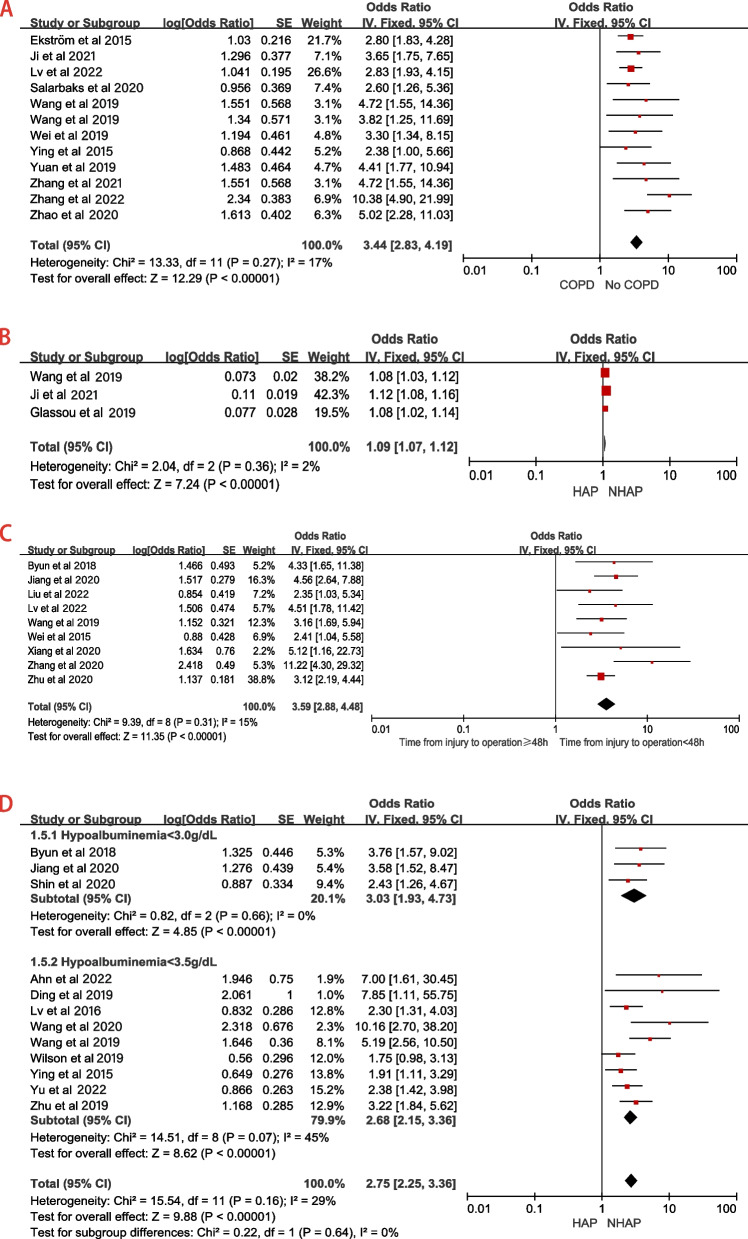
Forest plots for the other most frequently mentioned risk factors (> 10 articles). **a** Sensitivity analysis for COPD as a dichotomous variable; **b** Sensitivity analysis for time from injury to operation as a continuous variable (per hour increase); **c** Forest plot for time from injury to operation as a dichotomous variable (≥48 h vs. < 48 h); **d** Sensitivity analysis after subgroup analysis of hypoalbuminemia as a dichotomous variable (hypoalbuminemia< 3.0 g/L vs. ≥3.0 g/L and hypoalbuminemia< 3.5 g/L vs. ≥3.5 g/L)

Fourteen studies [4, 6, 9, 14, 19, 20, 23–25, 29, 31–34] reported the relationship between chronic obstructive pulmonary disease (COPD) and HAP (Table 2). Among these, 3 studies (Bohl, Salarbaks, Ying) reported a negative association between COPD and HAP, while the remaining 11 studies identified COPD as a risk factor for HAP. However, significant heterogeneity was observed among the pooled results (*P* < 0.001, I^2^ = 81%; in Supplementary eFigure2.A). To explore the potential sources of this heterogeneity, sensitivity analyses were conducted by systematically excluding each study and assessing its impact on the overall pooled estimates. Remarkably, when excluding the studies by Chen and Bohl, a significant reduction in between-study heterogeneity was observed (*P* = 0.27, I^2^ = 17%; Fig. 4A and Table 3). Hip fracture patients with COPD were 3.44 times more likely to have HAP than those without COPD (Fixed-effects model; OR 3.44, 95% CI 2.83–4.19; Fig. 4A and Table 3).

**Fig. 5:**
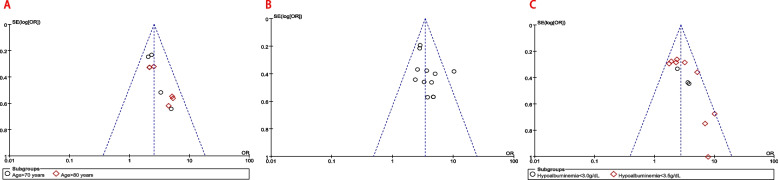
Funnel plots for the risk factors included ten or more studies. **a** Funnel plot for advanced age subgroup; **b** Funnel plot after sensitivity analysis for COPD; **c** Funnel plot after sensitivity analysis for hypoalbuminemia subgroup

A funnel plot for COPD was used to evaluate publication bias (Fig. 5B). Meanwhile, we performed Begg’s and Egger’s tests (in Supplementary eFigure2.B) for COPD. The results showed *P* > 0.05, indicating no publication bias for COPD.

### Patient factors - hypoalbuminemia

Fourteen studies [2, 11, 13, 16, 20, 22, 23, 28–30, 33, 35–37] reported the relationship between hypoalbuminemia (dichotomous variable) and HAP, of which four studies [2, 16, 22, 29] reported the association between hypoalbuminemia < 3.0 g/dL and HAP, and the other ten [11, 13, 20, 23, 28, 30, 33, 35–37] reported the relationship between hypoalbuminemia < 3.5 g/dL and HAP. Subgroup analysis was conducted for the 14 studies due to different levels among the studies. The results showed significant heterogeneity between the studies (*P* < 0.001, I^2^ = 68%; in Supplementary eFigure4. A). After sensitivity analysis, the heterogeneity between the studies decreased significantly (*P* = 0.16, I^2^ = 29%; Fig. 4D and Table 3). Further analysis showed that the lower the patient’s albumin level, the higher the incidence of HAP (hypoalbuminemia < 3.0 g/dL: OR = 3.03 vs. hypoalbuminemia < 3.5 g/dL: OR = 2.68; Fig. 4D and Table 3).

A Funnel plot for hypoalbuminemia (dichotomous variable) was used to evaluate publication bias (Fig. 5C). We also performed Begg’s and Egger’s tests (in Supplementary eFigure4.B&C). The results showed *P* > 0.05, indicating no publication bias among each subgroup.

### Treatment factor - time from injury to operation

Regarding the preoperative waiting time for hip fracture surgery, fifteen studies [16, 20–22, 24–27, 29, 33, 34, 38–41] reported the relationship between time from injury to operation and HAP (Table 2). Six studies [20, 25, 33, 34, 38, 39] reported the association between time from injury to operation (continuous variable) and HAP. The results showed significant heterogeneity among studies (*P* < 0.001, I^2^ = 93%; in Supplementary eFigure3). After Sensitivity analysis, the heterogeneity between the studies decreased significantly (*P* = 0.36, I^2^ = 2%; Fig. 4B and Table 3). The incidence of HAP increased 1.09 times every hour from injury to surgery (Fixed-effects model; OR 1.09, 95% CI 1.07–1.12; Fig. 4B and Table 3).

The remaining nine studies [16, 21, 22, 24, 26, 27, 29, 40, 41] reported the relationship between time from injury to operation (dichotomous variable: ≥48 h vs. < 48 h) and HAP. The results showed minor heterogeneity between the studies (*P* = 0.31, I^2^ = 15%; Fig. 4C and Table 3). Summarizing the results of these studies demonstrated that the incidence of HAP in patients with hip fractures who took more than 48 hours from injury to operation was 3.59 times higher than that in patients less than 48 hours (Fixed-effects model; OR 3.59, 95% CI 2.88–4.48; Fig. 4C and Table 3).

### Other factors

In addition to the above factors, we also analyzed nineteen other factors. There was heterogeneity in eleven factors, namely: male sex (*P* < 0.001, I^2^ = 78%), high BMI (*P* = 0.08, I^2^ = 55%), functional status (P < 0.001, I^2^ = 78%), CVA (*P* = 0.005, I^2^ = 81%), number of comorbidities (P < 0.001, I^2^ = 92%), type of anesthesia (*P* = 0.05, I^2^ = 52%), duration of surgery ≥2 h (*P* = 0.09, I^2^ = 59%), time of mechanical ventilation (*P* = 0.06, I^2^ = 71%), anemia (P < 0.001, I^2^ = 85%), and hyperglycemia (*P* = 0.007, I^2^ = 86%). After sensitivity analysis or subgroup analysis, the heterogeneity of nine factors has been resolved. Due to the small number of articles included by the time of mechanical ventilation, and hyperglycemia, the heterogeneity could not be solved.

In summary, among the 17 risk factors without heterogeneity issues, of which 15 factors were the risk factors for HAP in patients with hip fracture: male sex (OR 2.04, 95% CI 1.78–2.34), functional status-dependent (OR 3.13, 95% CI 2.11–4.63), history of smoking (OR 2.89, 95% CI 2.34–3.57), history of stroke (OR 3.10, 95% CI 2.28–4.20), CVA (OR 1.56, 95% CI 1.26–1.93), history of cancer (OR 3.77, 95% CI 2.13–6.67), cognitive function dysfunction (OR 2.75, 95% CI 1.86–4.07), number of comorbidities (OR 5.16, 95% CI 3.16–8.42), ASA ≥ 3 (OR 2.72, 95% CI 2.27–3.26), duration of surgery ≥2 h (OR 3.56, 95% CI 1.84–6.87), ICU (OR 2.92, 95% CI 1.93–4.41), extramedullary operation (OR 5.03, 95% CI 2.58–9.81), anemia (OR 2.97, 95% CI 2.14–4.11), high RDW (OR 3.14, 95% CI 2.35–4.20), and high Cr (OR 3.11, 95% CI 1.57–6.19). High BMI (OR 0.85, 95% CI 0.79–0.90) and intrathecal anesthesia (OR 0.24, 95% CI 0.18–0.32) were the protective factors. Detailed results can be found in Supplementary eFigure5–23 and Table 3. High BMI is a counterintuitive finding that requires further validation with increased sample size in the future. Recent articles have actively reported on the associations with history of stroke, type of anesthesia, and male sex. There is currently limited research on the correlation between HAP and hyperglycemia, as well as mechanical ventilation duration, making it a promising area for future exploration.

## Discussion

Hospital-acquired pneumonia (HAP) is a common complication in patients with hip fractures, with an incidence of 8.9% in our study, similar to the previously reported range of 4.0–9.0% [9, 35, 42]. In addition to the widely reported risk factors such as advanced age, COPD, time from injury to operation, and hypoalbuminemia, we also found that 17 other factors had statistical significance with HAP, including fifteen risk factors (Males, functional status-dependent, history of smoking, history of stroke, CVA, history of cancer, cognitive function dysfunction, number of comorbidities, ASA ≥ 3, duration of surgery ≥2 h, ICU, extramedullary operation, anemia, high RDW, and high Cr) and two protective factors (High BMI and intrathecal anesthesia). Therefore, a complete understanding and discussion of these risk factors were beneficial to reduce mortality and improving prognosis [6, 11, 17, 43].

Previous studies [2, 11, 23, 25] have suggested that advanced age (continuous variable) was an independent predictor of HAP, which was consistent with our research. However, the advanced age (dichotomous variable) definition varies among studies. To further assess the age cut-off for a significantly increased risk of HAP, we analyzed the age subgroups. Compared with other age groups, the probability of pneumonia occurring over 90 years old was increased considerably. This was related to the decline in the functions of various organs caused by aging [6, 18–20, 24, 28]. After the elderly hip fracture was bedridden, the tracheobronchial ciliary movement function weakened, the cough reflex worsened, the elasticity of lung tissue decreased, and the immunity of the elderly was weak, so pulmonary infection was easy to occur under long-term immobilization [4, 21, 44–46]. In the actual situation, advanced age as a single indicator to predict pneumonia is too single, and we should combine age with other factors for comprehensive analysis [18, 22, 29, 30]. Another critical factor was gender. Ekström et al. found that males were more than twice as likely as females to suffer from HAP [14]. Most studies believe this is caused by more disease exposure and a wider history of smoking in males than females [4, 6, 9, 36]. Therefore, smoking history was also a significant risk factor for HAP. In terms of patient BMI, we found that high BMI was a protective factor for HAP, which was interesting because high BMI in the past was associated with poor prognosis of patients [47, 48]. In this regard, Jiang and Byun et al. explained that the lower the BMI of patients, the higher the possibility of swallowing suffering, and the rate of aspiration will increase [16].

COPD is a significant risk factor for the occurrence and development of HAP. Lareau et al. found that due to the long-term impact of COPD, the structure and function of patients’ lungs and thorax changed, resulting in decreased compliance, imbalance of ventilation and blood flow, and irreversible lung injury [49]. Meanwhile, patients with a history of stroke, cancer, cardiovascular events, and cognitive function dysfunction can also significantly increase the incidence of HAP [14, 23, 28, 31–33]. Poole et al. believed that patients with hip fractures combined with stroke had decreased living ability to varying degrees and were prone to dysphagia and HAP, which required early intervention for protection [50]. In a nationwide cohort study, Søgaard et al. confirmed the correlation between cancer and HAP [51]. Cardiovascular events and cognitive function dysfunction caused and affected each other, and both acted on HAP [52–54]. In our study, we emphasized the subgroup analysis of the number of comorbidities. The results showed that the higher the number of comorbidities, the higher the incidence of HAP. When the Number of comorbidities ≥3, the incidence of HAP can be increased by 6.6 times. Combining age with the number of comorbidities as a concern value can improve the accuracy of the prediction of HAP [14, 29, 41].

In this study, the time from injury to surgery in the HAP group was significantly longer than in the non-HAP group. Some studies found that the probability of death, acute respiratory distress syndrome, myocardial infarction, and other complications of hip fracture patients who underwent surgery 48 hours after admission increased [21, 38–40]. Klestil et al. mentioned in a recent review that patients with complications can usually benefit from surgery within 24 hours [55]. Therefore, patients with hip fractures must be hospitalized as soon as possible to evaluate whether to carry out surgical treatment. If patients need surgical treatment, then the preoperative ASA score [11, 56], the type of anesthesia [26, 30], the type of operation [11, 25], duration of surgery [22, 27], and whether or not to enter ICU monitoring after surgery [20, 57] may increase the incidence of HAP. The specific mechanism varies from individual to individual. We also found that the mechanical ventilation time was related to HAP [20, 34]. However, there are few related studies, so the robustness of the results remains to be confirmed.

In terms of laboratory factors, hypoalbuminemia is often considered an important indicator of malnutrition and a common risk factor for surgical and inpatients [58, 59]. On one side, fracture healing and muscle recovery require much protein. When the protein is insufficient, it will lead to weakened limb function, affect fracture healing, and increase bed rest time; others, the deficiency of serum albumin causes the decrease of plasma colloid osmotic pressure and the increase of interstitial fluid, which may lead to pleural effusion, thus increasing the incidence of HAP [29, 60]. In contrast, high RDW and Cr levels are associated with HAP. When the RDW level is high, the number of mature red blood cells in the body decreases, which damages the blood microcirculation and reduces the tissues’ oxygen supply [61, 62]. The higher Cr level suggests the patient may have nephritis, leading to secondary pneumonia [63, 64]. Anemia may also cause the occurrence of HAP. Diet, chronic diseases, tumors, consumption after fracture, and blood leakage at the fracture site may all cause anemia in patients and increase the risk of HAP [6, 32]. Additionally, we found studies evaluating the relationship between hyperglycemia and HAP. Although there was no statistical significance between the two in this study, we believe this is because fewer studies were included [17, 30]. Rueda et al. have demonstrated that poor blood glucose control increases the risk of pneumonia [65]. We are looking forward to more high-quality studies in the future to confirm the relationship between hyperglycemia and HAP.

This study has the following notable strengths: First, this is the meta-analysis on risk factors of HAP in patients with hip fractures. Second, compared with the previous meta-analysis of risk factors, we have retrieved more databases and included more articles. Third, the inclusion of a substantial amount of Asian literature for the first time has addressed the potential influence of racial genetics, reducing the risks associated with regional bias and facilitating the generalization of research findings.

Nevertheless, this study has several limitations: Firstly, Significant heterogeneity was found in selected studies. Secondly, Only the factors after multivariate logistic regression are included. Although this improves the study’s accuracy, it will cause some factors related to HAP not to be included. Thirdly, randomized controlled trials (RCTs) were not included in order to align with the design of this study. Consequently, this has significantly impacted the level of evidence available to us. Fourthly, the exclusion of other languages has resulted in certain limitations in our study. Finally, the lack of diversity within the study population included in this research significantly undermines the generalizability and external validity of the findings.

## Conclusions

In conclusion, this meta-analysis of 35 articles and 337,818 patients comprehensively assessed the incidence and risk factors for hospital-acquired pneumonia (HAP) in patients with hip fractures. The study found that HAP had an incidence of 8.9% and identified 21 significant risk factors, including advanced age, COPD, hypoalbuminemia, male gender, functional status-dependent, history of smoking, history of stroke, CVA, history of cancer, cognitive function dysfunction, number of comorbidities, ASA ≥ 3, duration of surgery ≥2 h, ICU, extramedullary operation, anemia, high RDW, and high Cr. These findings can help clinicians identify patients at risk of HAP and implement preventive measures to reduce the incidence of this devastating complication during hospitalization. Furthermore, modifiable laboratory indicators such as albumin levels will serve as key factors for future researchers to focus on in mechanistic studies.

### Supplementary Information

**Additional file 1: **Appendix. eFigures 1-23 and eTable 1.

**Additional file 2: **Search strategies.

**Additional file 3: **PRISMA 2020 flow diagram for new systematic reviews which included searches of databases, registers and other sources.

**Additional file 4: **PRISMA 2020 Checklist.
