# Supplementary material for: Risk factors for hospital-acquired pneumonia in hip fracture patients: a systematic review and meta-analysis
Source: BMC Musculoskelet Disord. 2024 Jan 2;25:6. doi: 10.1186/s12891-023-07123-0 (PMC10759764; doi:10.1186/s12891-023-07123-0)
Supplement: Supplementary file 1 — Additional file 1: Appendix. eFigures 1-23 and eTable 1. [file 12891_2023_7123_MOESM1_ESM.pdf]

Appendix

A

Study or Subgroup	log[Odds Ratio]	SE	Weight	Odds Ratio IV, Random, 95% CI
Zhu et al 2019	0.064	0.021	21.4%	1.07 [1.02, 1.11]
Ying et al 2015	0.568	0.287	0.3%	1.76 [1.01, 3.10]
Wei et al 2019	0.113	0.025	18.6%	1.12 [1.07, 1.18]
Shin et al 2020	0.06	0.019	23.0%	1.06 [1.02, 1.10]
Lv et al 2016	0.494	0.181	0.8%	1.64 [1.15, 2.34]
Jiang et al 2020	0.06	0.026	18.0%	1.06 [1.01, 1.12]
Byun et al 2018	0.072	0.026	18.0%	1.07 [1.02, 1.13]
**Total (95% CI)**			**100.0%**	**1.08 [1.05, 1.12]**

Heterogeneity: Tau² = 0.00; Chi² = 11.84, df = 6 (P = 0.07); I² = 49%
Test for overall effect: Z = 4.83 (P < 0.00001)

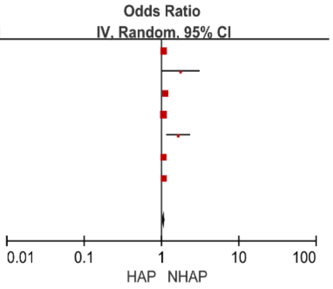

B

Age>70 years

```
Tests for Publication Bias

Begg's Test

  adj. Kendall's Score (P-Q) =       6
          Std. Dev. of Score =    4.08
           Number of Studies =       5
                          z  =    1.47
                  Pr > |z|   =   0.142
                          z  =    1.22 (continuity corrected)
                  Pr > |z|   =   0.221 (continuity corrected)
```

Egger's test

Std_Eff	Coefficient	Std. err.	t	P>|t|	[95% conf.	interval]
slope	.3436029	.1663539	2.07	0.131	-.1858094	.8730152
bias	1.699432	.5249196	3.24	0.048	.0289035	3.36996

C

Age>80 years

```
Tests for Publication Bias

Begg's Test

  adj. Kendall's Score (P-Q) =       4
          Std. Dev. of Score =    4.08
           Number of Studies =       5
                          z  =    0.98
                  Pr > |z|   =   0.327
                          z  =    0.73 (continuity corrected)
                  Pr > |z|   =   0.462 (continuity corrected)
```

Egger's test

Std_Eff	Coefficient	Std. err.	t	P>|t|	[95% conf.	interval]
slope	-.0828588	.2483457	-0.33	0.761	-.8732057	.7074881
bias	2.902481	.5889198	4.93	0.016	1.028275	4.776686

eFigure 1 Forest plots and publication bias tests for advanced age. **A.** Forest plot for advanced age (Continuous); **B&C.** Publication bias test for subgroup analysis of advanced age (Dichotomous).

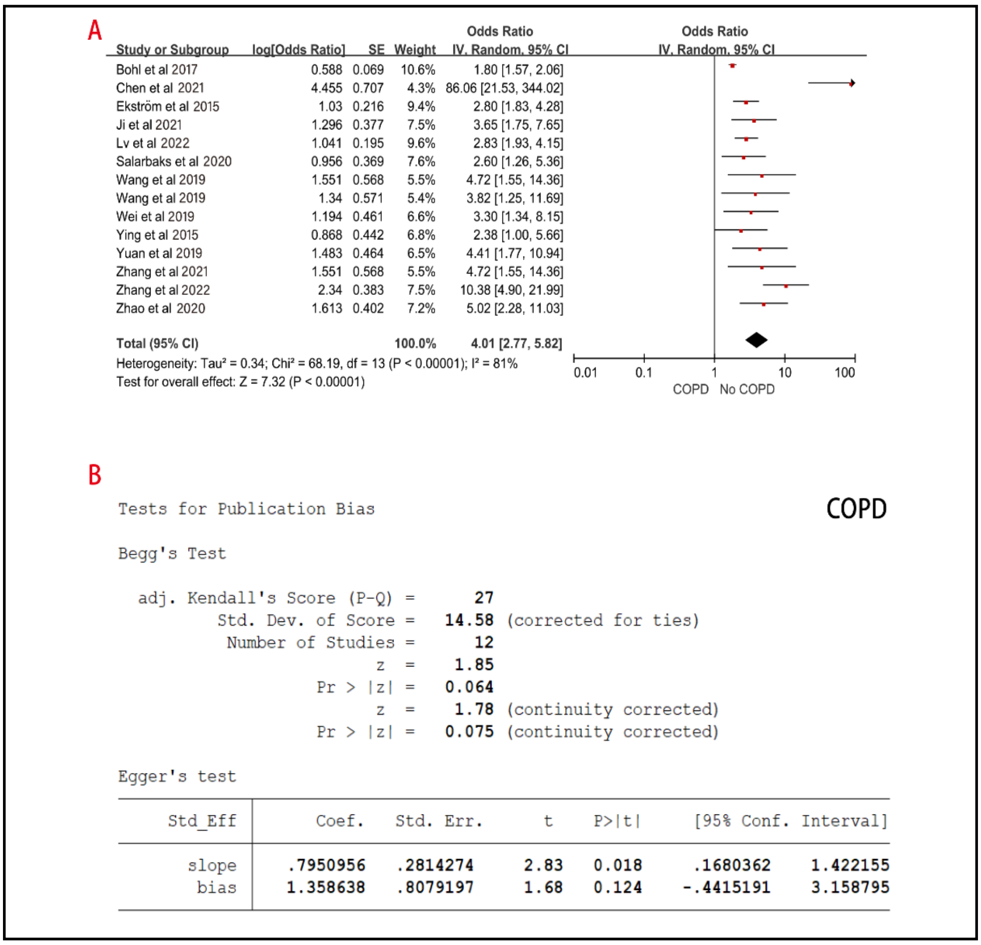

A

Study or Subgroup	log[Odds Ratio]	SE	Weight	Odds Ratio IV, Random, 95% CI
Bohl et al 2017	0.588	0.069	10.6%	1.80 [1.57, 2.06]
Chen et al 2021	4.455	0.707	4.3%	86.06 [21.53, 344.02]
Ekström et al 2015	1.03	0.216	9.4%	2.80 [1.83, 4.28]
Ji et al 2021	1.296	0.377	7.5%	3.65 [1.75, 7.65]
Lv et al 2022	1.041	0.195	9.6%	2.83 [1.93, 4.15]
Salarbaks et al 2020	0.956	0.369	7.6%	2.60 [1.26, 5.36]
Wang et al 2019	1.551	0.568	5.5%	4.72 [1.55, 14.36]
Wang et al 2019	1.34	0.571	5.4%	3.82 [1.25, 11.69]
Wei et al 2019	1.194	0.461	6.6%	3.30 [1.34, 8.15]
Ying et al 2015	0.868	0.442	6.8%	2.38 [1.00, 5.66]
Yuan et al 2019	1.483	0.464	6.5%	4.41 [1.77, 10.94]
Zhang et al 2021	1.551	0.568	5.5%	4.72 [1.55, 14.36]
Zhang et al 2022	2.34	0.383	7.5%	10.38 [4.90, 21.99]
Zhao et al 2020	1.613	0.402	7.2%	5.02 [2.28, 11.03]
**Total (95% CI)**			**100.0%**	**4.01 [2.77, 5.82]**

Heterogeneity: Tau² = 0.34; Chi² = 68.19, df = 13 (P < 0.00001); I² = 81%
Test for overall effect: Z = 7.32 (P < 0.00001)

B

COPD

```
Tests for Publication Bias

Begg's Test

  adj. Kendall's Score (P-Q) =      27
          Std. Dev. of Score =   14.58 (corrected for ties)
           Number of Studies =      12
                           z =    1.85
                    Pr > |z| =   0.064
                           z =    1.78 (continuity corrected)
                    Pr > |z| =   0.075 (continuity corrected)

Egger's test
```

Std_Eff	Coef.	Std. Err.	t	P>|t|	[95% Conf.	Interval]
slope	.7950956	.2814274	2.83	0.018	.1680362	1.422155
bias	1.358638	.8079197	1.68	0.124	-.4415191	3.158795

eFigure 2 Forest plots and publication bias tests for COPD (Dichotomous). **A.** Forest plot for COPD; **B.** Publication bias test for COPD.

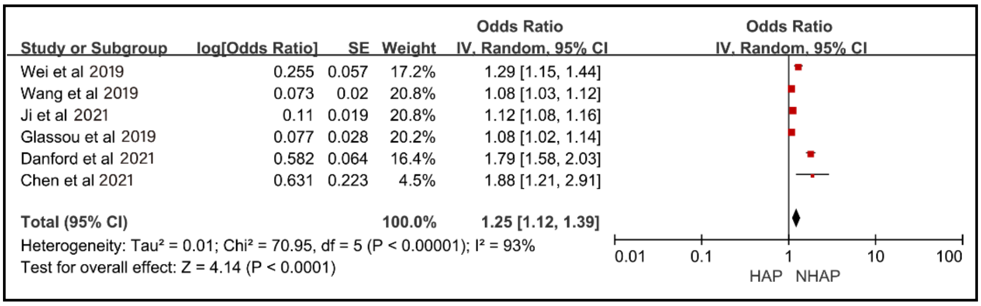

Study or Subgroup	log[Odds Ratio]	SE	Weight	Odds Ratio IV, Random, 95% CI
Wei et al 2019	0.255	0.057	17.2%	1.29 [1.15, 1.44]
Wang et al 2019	0.073	0.02	20.8%	1.08 [1.03, 1.12]
Ji et al 2021	0.11	0.019	20.8%	1.12 [1.08, 1.16]
Glassou et al 2019	0.077	0.028	20.2%	1.08 [1.02, 1.14]
Danford et al 2021	0.582	0.064	16.4%	1.79 [1.58, 2.03]
Chen et al 2021	0.631	0.223	4.5%	1.88 [1.21, 2.91]
**Total (95% CI)**			**100.0%**	**1.25 [1.12, 1.39]**

Heterogeneity: Tau² = 0.01; Chi² = 70.95, df = 5 (P < 0.00001); I² = 93%
Test for overall effect: Z = 4.14 (P < 0.0001)

eFigure 3 Forest plot for time from injury to operation (Continuous).

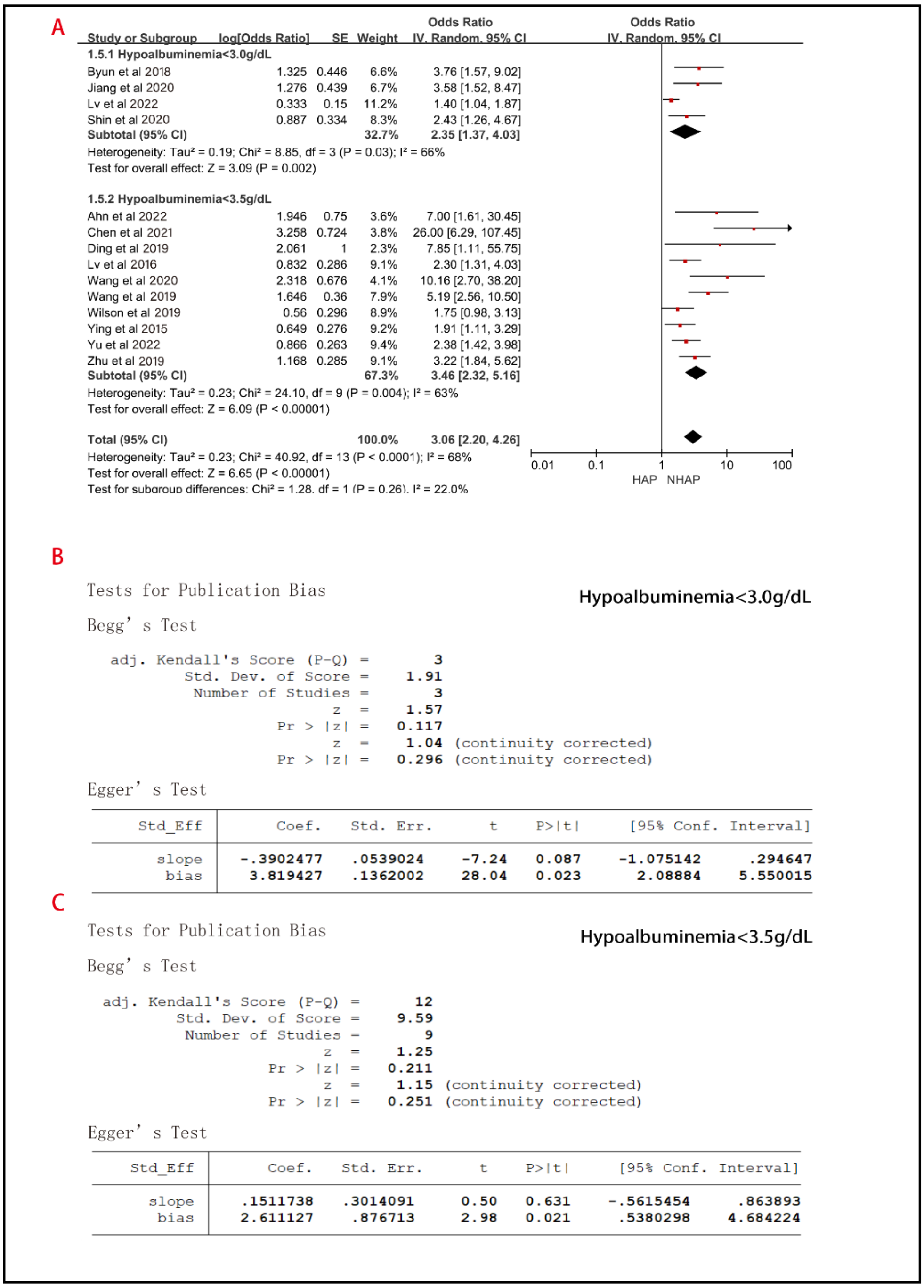

eFigure 4 Forest plots and publication bias tests for hypoalbuminemia (Dichotomous). **A.** Forest plot for subgroup Analysis; **B&C.** Publication bias test for subgroup analysis

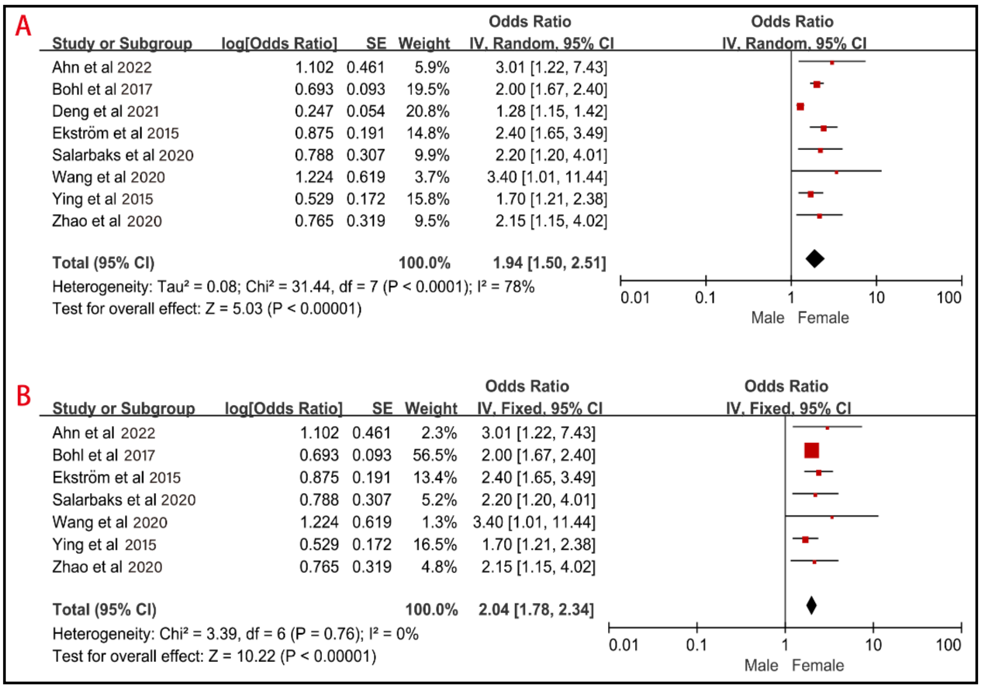

eFigure 5 Forest plots and sensitivity analysis for male sex (Dichotomous). **A.** Forest plot for male sex; **B.** Sensitivity analysis for male sex.

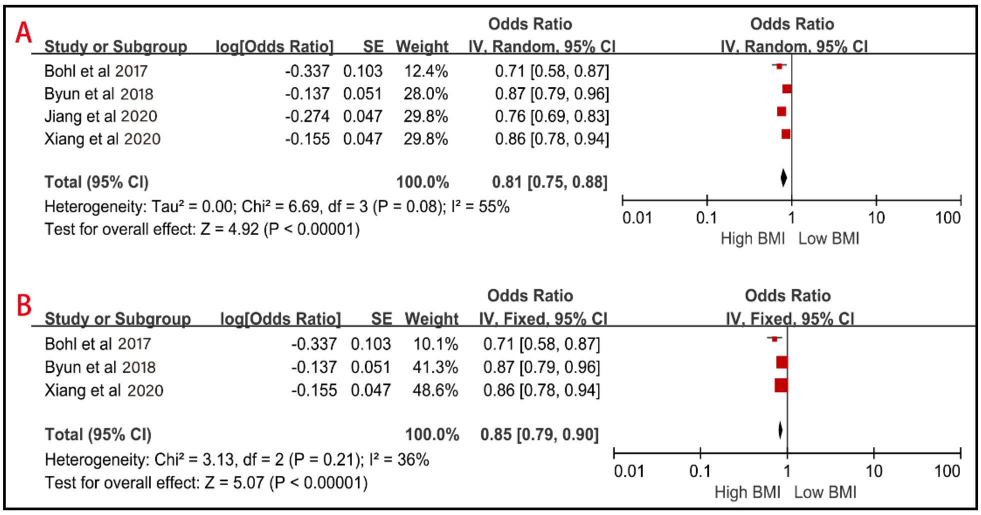

eFigure 6 Forest plots and sensitivity analysis for High BMI (Continuous). **A.** Forest plot for High BMI; **B.** Sensitivity analysis for High BMI.

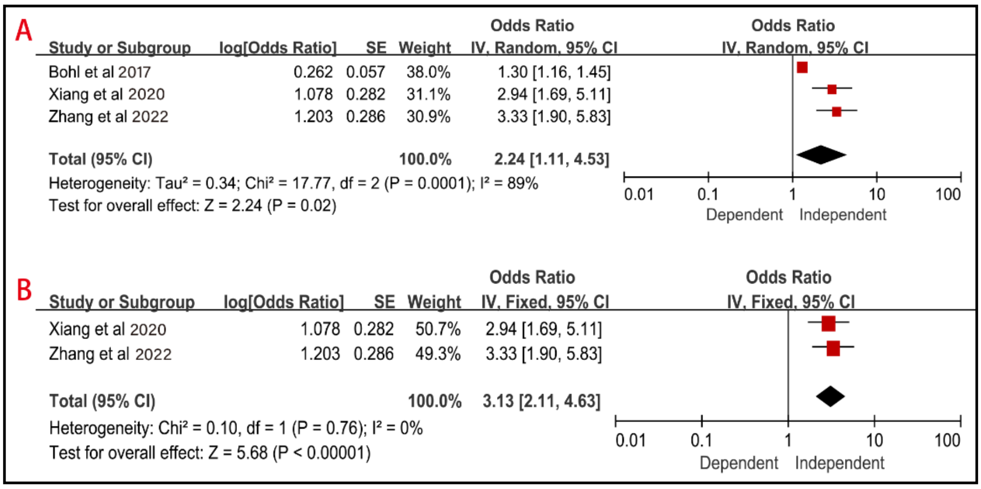

eFigure 7 Forest plots and sensitivity analysis for functional status (Dichotomous). **A.** Forest plot for functional status; **B.** Sensitivity analysis for functional status.

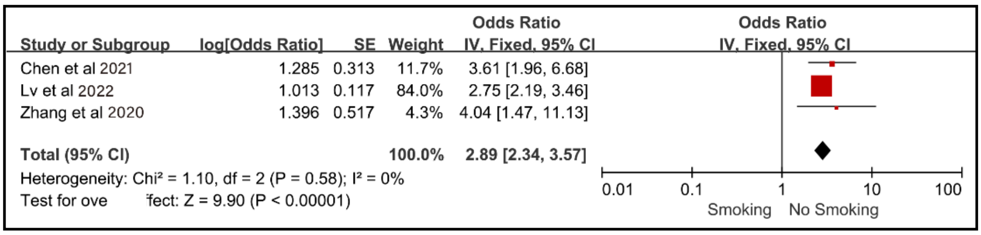

eFigure 8 Forest plot for the history of smoking (Dichotomous).

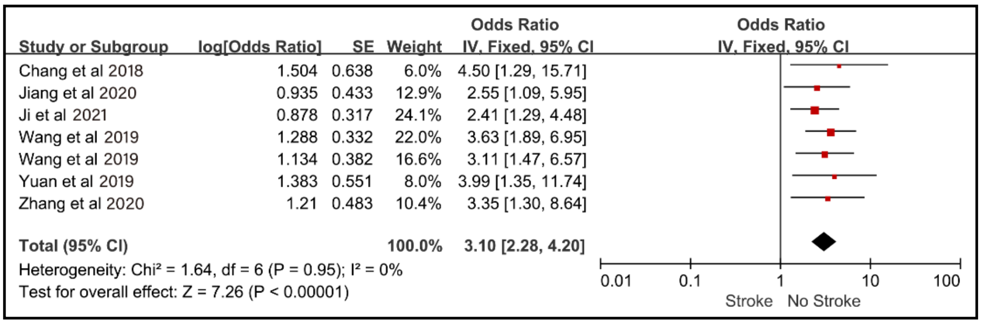

eFigure 9 Forest plot for the history of stroke (Dichotomous).

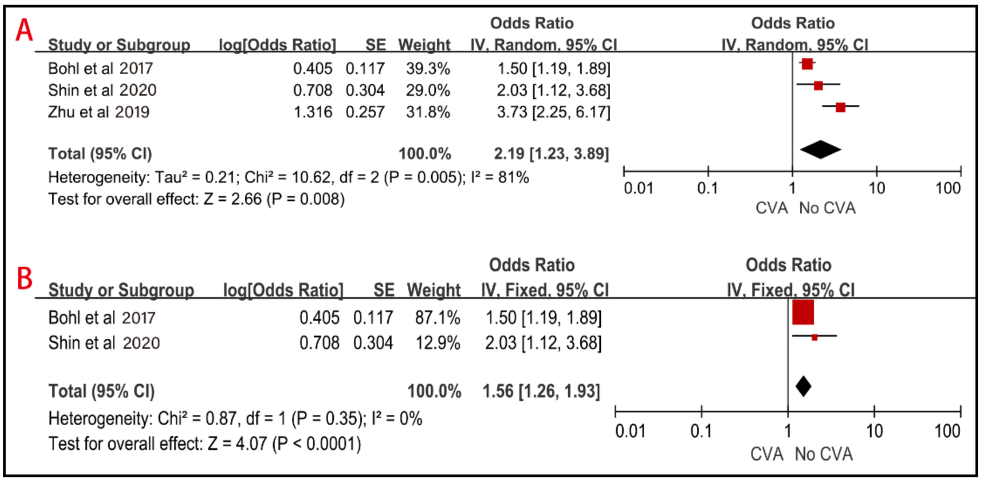

eFigure 10 Forest plots and sensitivity analysis for CVA (Dichotomous). A. Forest plot for CVA; B. Sensitivity analysis for CVA.

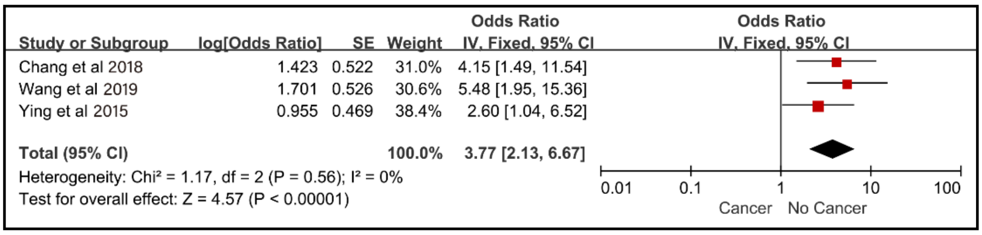

eFigure 11 Forest plot for the history of cancer (Dichotomous).

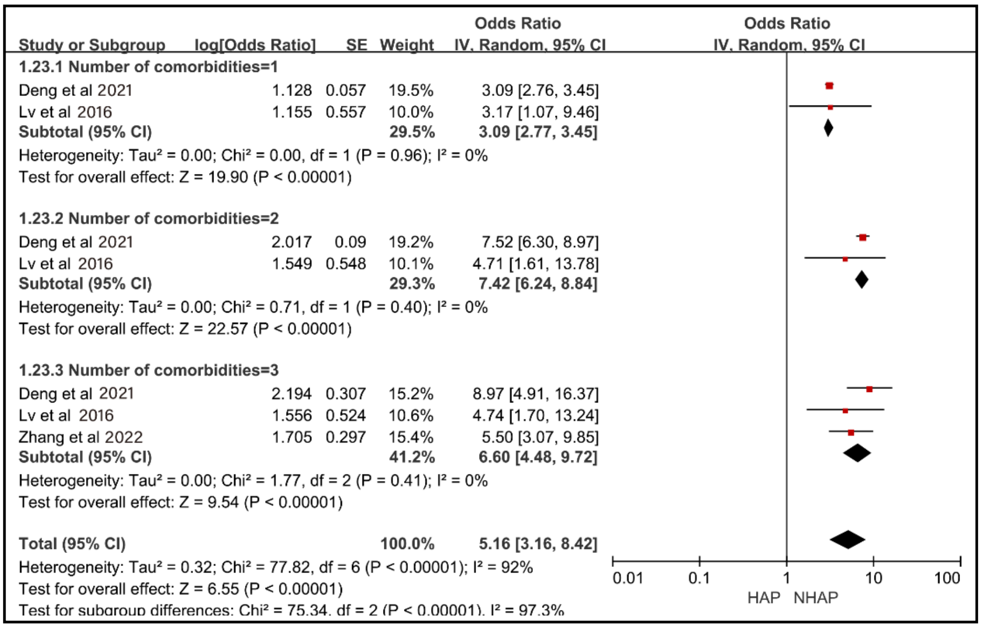

eFigure 12 Forest plot for subgroup Analysis of Number of comorbidities (Stratification).

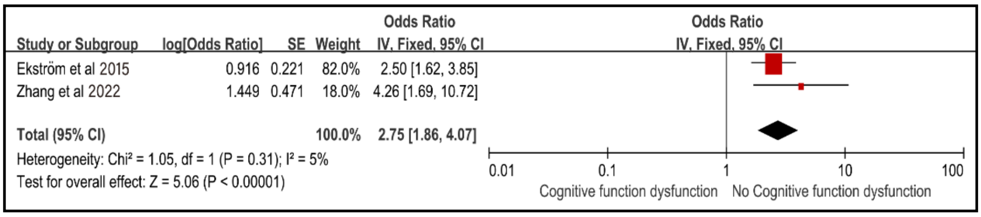

eFigure 13 Forest plot for cognitive function dysfunction (Dichotomous).

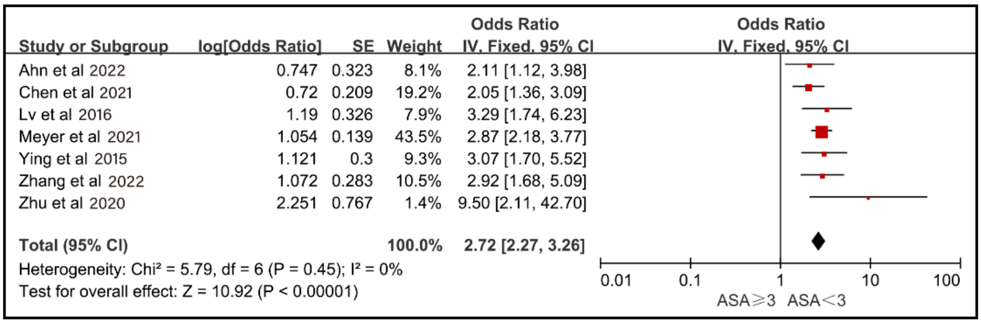

eFigure 14 Forest plot for ASA (Dichotomous).

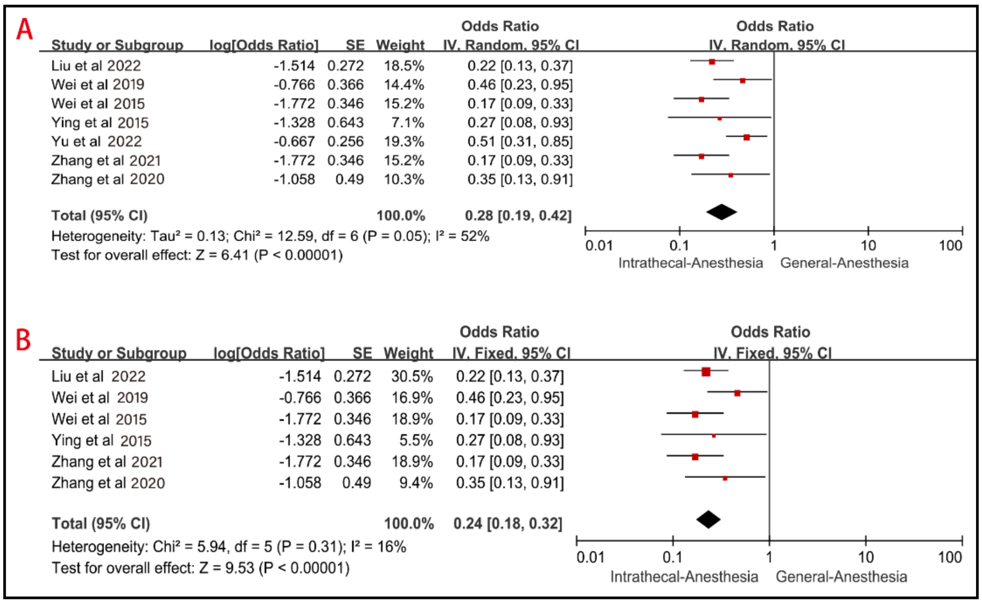

Figure 15 Forest plots and sensitivity analysis for the type of anesthesia (Dichotomous). **A.** Forest plot for the type of anesthesia; **B.** Sensitivity analysis for the type of anesthesia.

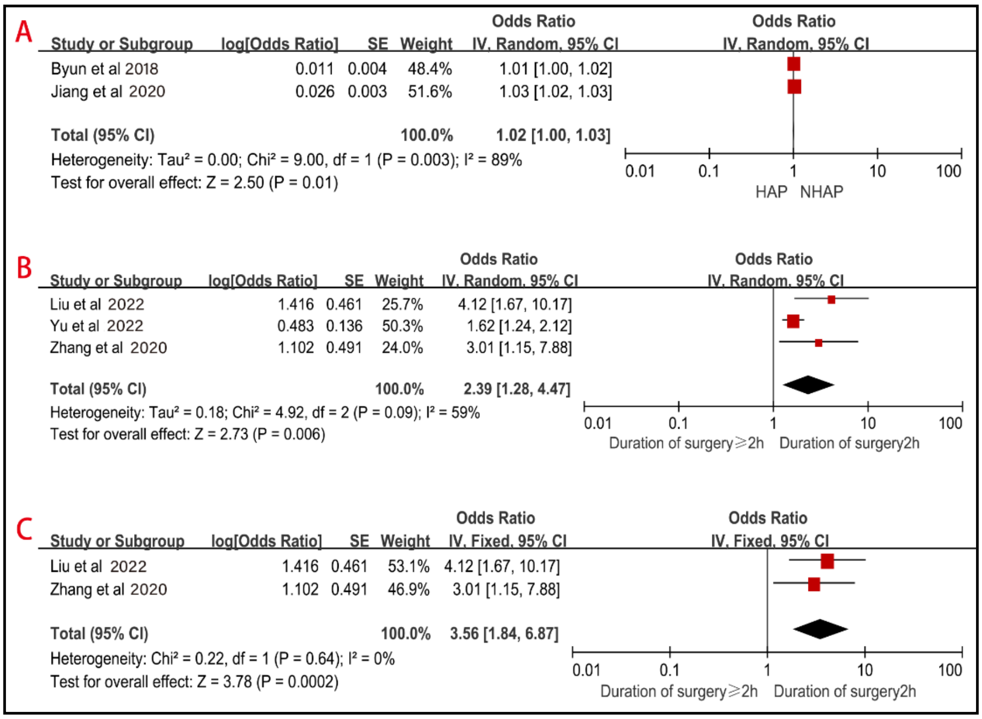

eFigure 16 Forest plots and sensitivity analysis for the duration of surgery. A. Forest plot for the duration of surgery (Continuous); B. Forest plot for the duration of surgery (Dichotomous); C. Sensitivity analysis for the duration of surgery (Dichotomous).

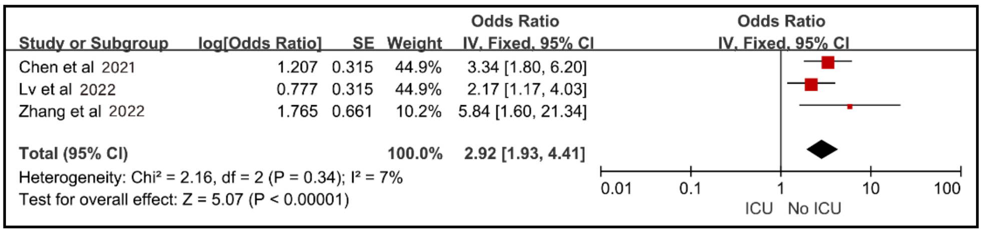

eFigure 17 Forest plot for ICU (Dichotomous).

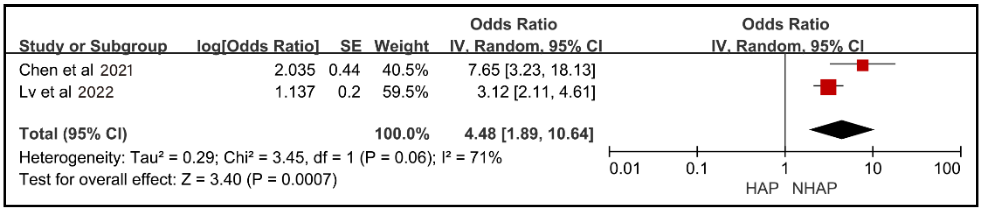

eFigure 18 Forest plot for the time of Mechanical ventilation (Continuous).

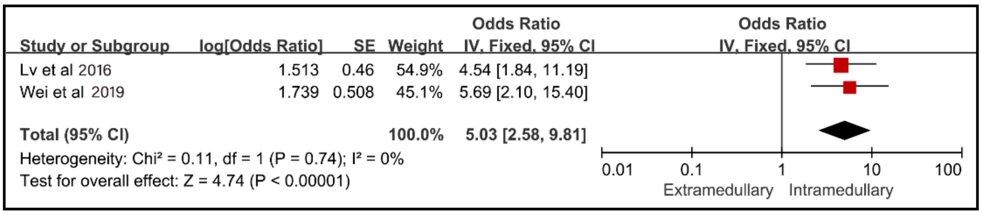

eFigure 19 Forest plot for the type of operation (Dichotomous).

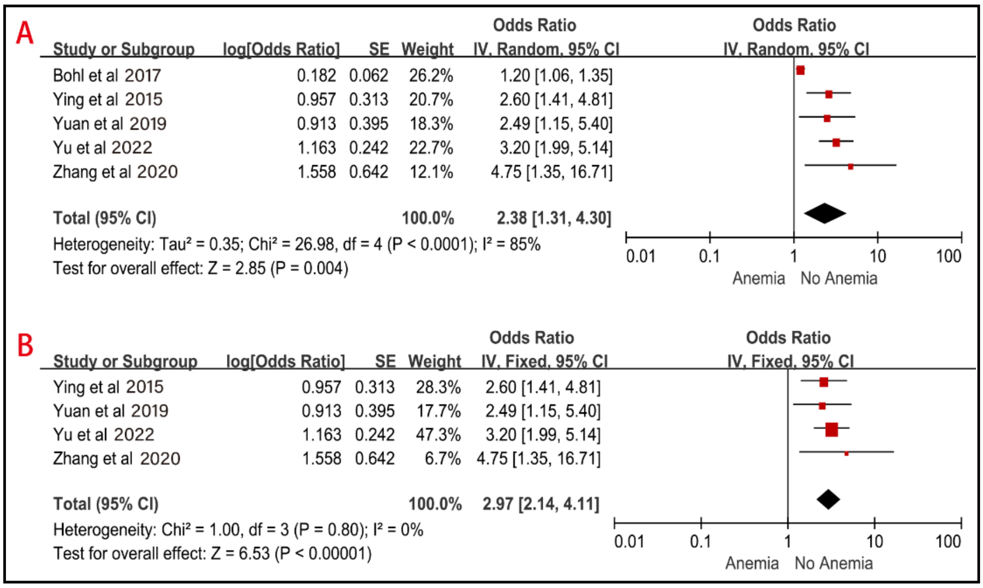

Figure 20 Forest plots and sensitivity analysis for anemia (Dichotomous). **A.** Forest plot for anemia; **B.** Sensitivity analysis for anemia.

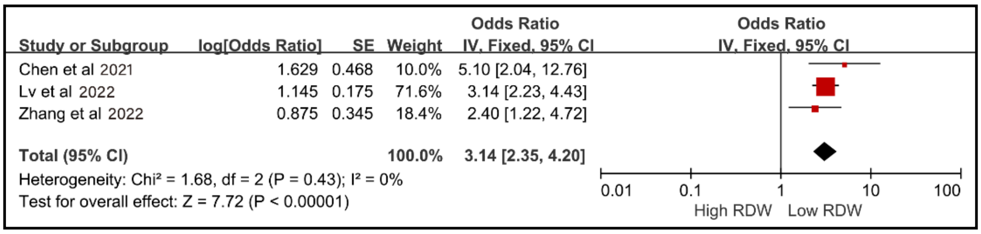

eFigure 21 Forest plot for High RDW (Continuous).

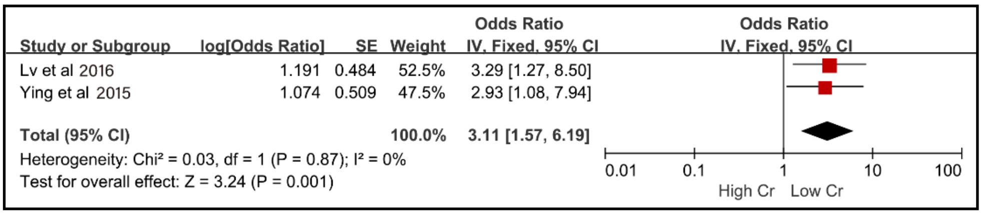

eFigure 22 Forest plot for High Cr (Continuous).

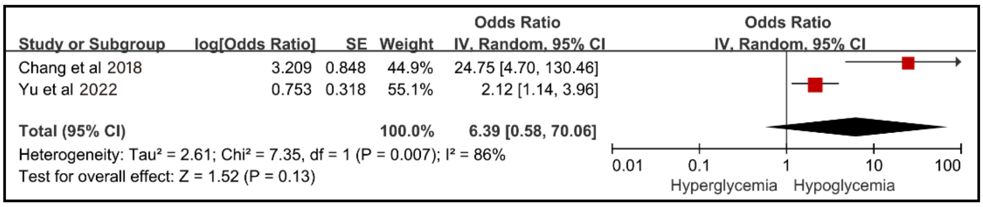

eFigure 23 Forest plot for hyperglycemia (Dichotomous).

eTable 1 Quality assessment of included studies

NOS scale									
Study	Selection				Comparability 2 stars	outcomes			Total 9 star
	Representativeness of the exposed cohort 1 star	Selection of the nonexposed cohort 1 star	Ascertainment of exposure 1 star	Demonstration that outcome of interest was not present at start of study 1 star		Assessment of outcome 1 star	Follow-up long enough for outcomes to occur 1 star	Adequacy of follow up of cohort 1 star	
Bohl et al. 2017	★	★	★	★	★★	★	★		8
Wilson et al. 2019	★	★	★	★	★★	★			7
Danford et al. 2021	★	★	★	★	★★	★			7
Ekström et al. 2015	★	★	★	★	★★	★	★	★	9
Meyer et al. 2021	★	★	★	★	★★	★	★	★	9
Salarbaks et al. 2020	★	★	★	★	★★	★	★		8
Kim et al. 2017	★	★	★	★	★★	★	★		8
Byun et al. 2018	★	★	★	★	★★	★	★		8
Shin et al. 2020	★	★	★	★	★★	★	★		8
Ahn et al. 2022	★	★	★	★	★★	★	★		8
Glassou et al. 2019	★	★	★	★	★★	★	★		8
Pagotto et al. 2022	★	★	★	★	★★	★	★	★	9
Chang et al. 2018	★	★	★	★	★★	★	★		8
Deng et al. 2021	★	★	★	★	★★	★	★	★	9
Shen et al. 2021	★	★	★	★	★★	★			7

Wang et al. 2020	★	★	★	★	★★	★	★	★	9
Xiang et al. 2020	★	★	★	★	★★	★	★		8
Zhang et al. 2021	★	★	★	★	★★	★	★		8
Zhang et al. 2022	★	★	★	★	★★	★	★	★	9
Chen et al. 2021	★	★	★	★	★★	★	★	★	9
Ding et al. 2019	★	★	★	★	★★	★	★		8
Liu et al. 2022	★	★	★		★★	★	★		7
Jiang et al. 2020	★	★	★	★	★★	★	★		8
Ying et al. 2015	★	★	★	★	★★	★	★		8
Li et al. 2014	★	★			★	★			4
Yuan et al. 2020	★	★			★★	★			5
Wang et al. 2019	★	★	★		★★	★	★		7
Wei et al. 2019	★	★		★	★★	★	★		7
Wei et al. 2015	★	★	★		★★	★	★		7
Huang et al. 2014	★	★			★★	★			5
Zhang et al. 2020	★	★	★	★	★★	★	★	★	9
Zhu et al. 2019	★	★	★		★★	★	★		7
Yuan et al. 2019	★	★		★	★★	★	★		7
Zhu et al. 2020	★	★	★	★	★★	★	★		8
Lv et al. 2016	★	★	★	★	★★	★	★	★	9
Wang et al.	★	★			★	★			4

2019									
Ye et al. 2012	★	★			★	★			4
Chen et al. 2022	★	★	★		★	★			5
Wang et al. 2020	★	★	★	★	★★	★	★	★	9
Zhao et al. 2020	★	★	★		★★	★	★		7
Ji et al. 2021	★	★	★	★	★★	★	★	★	9
Lv et al. 2022	★	★	★	★	★★	★	★		8
Yu et al. 2022	★	★	★	★	★★	★	★	★	9
Zhang et al. 2022	★	★	★	★	★★	★	★	★	9

*NOS: Newcastle–Ottawa Scale.
