# Supplementary material for: Risk factors for hospital-acquired pneumonia in hip fracture patients: a systematic review and meta-analysis
Source: BMC Musculoskelet Disord. 2024 Jan 2;25:6. doi: 10.1186/s12891-023-07123-0 (PMC10759764; doi:10.1186/s12891-023-07123-0)
Supplement: Supplementary file 2 — Additional file 2: Search strategies. [file 12891_2023_7123_MOESM2_ESM.docx]

**Search strategies**

| **SinoMed** | ("相关因素"[常用字段] OR "病因"[常用字段] OR "影响因素"[常用字段] OR "危险因素"[不加权:扩展]) AND (("股骨头骨折"[常用字段] OR "股骨粗隆下骨折"[常用字段] OR "股骨粗隆间骨折"[常用字段] OR "髋骨折"[常用字段] OR "股骨颈骨折"[不加权:扩展] OR "转子下骨折"[不加权:扩展] OR "转子间骨折"[不加权:扩展]) AND (("肺炎链球菌"[常用字段] OR "肺炎双球菌"[常用字段] OR "肺炎球菌"[常用字段] OR "肺炎链球菌"[主题词]) OR ("肺疾病"[常用字段] OR "肺部疾病"[常用字段] OR "肺疾病"[主题词] OR "肺部感染"[常用字段]) OR ("肺炎球菌"[常用字段] OR "肺炎链球菌"[常用字段] OR "肺炎双球菌"[常用字段] OR "肺炎链球菌"[主题词]) OR ("胸膜肺炎"[常用字段] OR "胸膜肺炎"[主题词]) OR "肺炎"[不加权:扩展])) |
| --- | --- |
| **CNKI** | ( ( ( (SU%=股骨颈骨折 OR TI=股骨颈骨折 OR v_subject=中英文扩展(股骨颈骨折) OR title=中英文扩展(股骨颈骨折)) OR (SU%=髋部骨折 OR TI=髋部骨折 OR v_subject=中英文扩展(髋部骨折) OR title=中英文扩展(髋部骨折)) ) OR ( (SU%=股骨粗隆间骨折 OR TI=股骨粗隆间骨折 OR v_subject=中英文扩展(股骨粗隆间骨折) OR title=中英文扩展(股骨粗隆间骨折)) OR (SU%=股骨粗隆下骨折 OR TI=股骨粗隆下骨折 OR v_subject=中英文扩展(股骨粗隆下骨折) OR title=中英文扩展(股骨粗隆下骨折)) ) ) OR (SU%=转子间骨折 OR TI=转子间骨折 OR v_subject=中英文扩展(转子间骨折) OR title=中英文扩展(转子间骨折)) OR (SU%=转子下骨折 OR TI=转子下骨折 OR v_subject=中英文扩展(转子下骨折) OR title=中英文扩展(转子下骨折)) OR (SU%=股骨头骨折 OR TI=股骨头骨折 OR v_subject=中英文扩展(股骨头骨折) OR title=中英文扩展(股骨头骨折)) ) AND ( ( (AB=危险因素 OR abstract_en=中英文扩展(危险因素)) OR (AB=影响因素 OR abstract_en=中英文扩展(影响因素)) ) OR ( (AB=病因 OR abstract_en=中英文扩展(病因)) OR (AB=相关因素 OR abstract_en=中英文扩展(相关因素)) ) ) AND ( ( ( (SU%=肺炎 OR TI=肺炎 OR v_subject=中英文扩展(肺炎) OR title=中英文扩展(肺炎)) OR (SU%=胸膜肺炎 OR TI=胸膜肺炎 OR v_subject=中英文扩展(胸膜肺炎) OR title=中英文扩展(胸膜肺炎)) ) OR ( (SU%=肺炎球菌 OR TI=肺炎球菌 OR v_subject=中英文扩展(肺炎球菌) OR title=中英文扩展(肺炎球菌)) OR (SU%=肺疾病 OR TI=肺疾病 OR v_subject=中英文扩展(肺疾病) OR title=中英文扩展(肺疾病)) ) ) OR (SU%=肺部感染 OR TI=肺部感染 OR v_subject=中英文扩展(肺部感染) OR title=中英文扩展(肺部感染)) OR (SU%=肺炎链球菌 OR TI=肺炎链球菌 OR v_subject=中英文扩展(肺炎链球菌) OR title=中英文扩展(肺炎链球菌)) ) (模糊匹配) |
| **WAN FANG** | 主题:(股骨颈骨折 or 髋部骨折 or 股骨粗隆间骨折 or 股骨粗隆下骨折 or 股骨头骨折 or 转子下骨折 or 转子间骨折) and 主题:(肺炎 or 胸膜肺炎 or 肺炎球菌 or 肺疾病 or 肺炎链球菌 or 肺部感染) and 主题:(危险因素 or 影响因素 or 病因 or 相关因素) |
| **CQVIP** | (M=股骨颈骨折 or 髋部骨折 or 股骨粗隆间骨折 or 股骨粗隆下骨折 or 股骨头骨折 or 转子间骨折 or 转子下骨折) AND (M=肺炎 or 胸膜肺炎 or 肺炎球菌 or 肺疾病 or 肺炎链球菌 or 肺部感染) AND (M=危险因素 or 影响因素 or 病因 or 相关因素) |
| **PubMed** | ((("Pneumonia"[Mesh]) OR ((((((((((((((((((((Lobar Pneumonia[Title/Abstract]) OR (Pneumonias[Title/Abstract])) OR (Lobar Pneumonias[Title/Abstract])) OR (Pneumonias, Lobar[Title/Abstract])) OR (Pneumonia, Lobar[Title/Abstract])) OR (Experimental Lung Inflammation[Title/Abstract])) OR (Experimental Lung Inflammations[Title/Abstract])) OR (Inflammation, Experimental Lung[Title/Abstract])) OR (Lung Inflammation, Experimental[Title/Abstract])) OR (Lung Inflammations, Experimental[Title/Abstract])) OR (Pneumonitis[Title/Abstract])) OR (Pneumonitides[Title/Abstract])) OR (Pulmonary Inflammation[Title/Abstract])) OR (Inflammation, Pulmonary[Title/Abstract])) OR (Inflammations, Pulmonary[Title/Abstract])) OR (Pulmonary Inflammations[Title/Abstract])) OR (Lung Inflammation[Title/Abstract])) OR (Inflammation, Lung[Title/Abstract])) OR (Inflammations, Lung[Title/Abstract])) OR (Lung Inflammations[Title/Abstract]))) AND ((("Hip Fractures"[Mesh]) OR ("Femoral Neck Fractures"[Mesh])) OR ((((((((((Fractures, Hip[Title/Abstract]) OR (Trochanteric Fractures[Title/Abstract])) OR (Fractures, Trochanteric[Title/Abstract])) OR (Intertrochanteric Fractures[Title/Abstract])) OR (Fractures, Intertrochanteric[Title/Abstract])) OR (Subtrochanteric Fractures[Title/Abstract])) OR (Fractures, Subtrochanteric[Title/Abstract])) OR (Femoral Neck Fracture[Title/Abstract])) OR (Femur Neck Fractures[Title/Abstract])) OR (Femur Neck Fracture[Title/Abstract])))) AND (((((((((((((relative[Title/Abstract]) OR (risk[Title/Abstract])) OR (relative risk[Title/Abstract])) OR (risks[Title/Abstract])) OR (association[Title/Abstract])) OR (Influencing factors[Title/Abstract])) OR (risk factors[Title/Abstract])) OR (risk factor[Title/Abstract])) OR (Cause[Title/Abstract])) OR (relevant factors[Title/Abstract])) OR (mortality[Title/Abstract])) OR (mortality[MeSH:noexp])) OR (risk[MeSH:noexp])) |
| **Cochrane Library** | #1 MeSH descriptor: [Hip Fractures] explode all trees 1836  #2 (Fractures, Hip):ab,ti,kw OR (Trochanteric Fractures):ab,ti,kw OR (Fractures, Trochanteric):ab,ti,kw OR (Intertrochanteric Fractures):ab,ti,kw OR (Fractures, Intertrochanteric):ab,ti,kw OR (Subtrochanteric Fractures):ab,ti,kw OR (Fractures, Subtrochanteric):ab,ti,kw 4572  #3 #1 or #2 4702  #4 MeSH descriptor: [Femoral Neck Fractures] explode all trees 462  #5 (Femoral Neck Fracture):ab,ti,kw OR (Femur Neck Fractures):ab,ti,kw OR (Femur Neck Fracture):ab,ti,kw 2367  #6 #4 or #5 2432  #7 #3 or #6 5864  #8 MeSH descriptor: [Pneumonia] explode all trees 5873  #9 (Pneumonias):ab,ti,kw OR (Lobar Pneumonia):ab,ti,kw OR (Lobar Pneumonias):ab,ti,kw OR (Pneumonias, Lobar):ab,ti,kw OR (Pneumonia, Lobar):ab,ti,kw OR (Experimental Lung Inflammation):ab,ti,kw OR (Experimental Lung Inflammations):ab,ti,kw OR (Inflammation, Experimental Lung):ab,ti,kw OR (Lung Inflammation, Experimental):ab,ti,kw OR (Lung Inflammations, Experimental):ab,ti,kw OR (Pneumonitis):ab,ti,kw OR (Pneumonitides):ab,ti,kw OR (Pulmonary Inflammation):ab,ti,kw OR (Inflammations, Pulmonary):ab,ti,kw OR (Inflammation, Pulmonary):ab,ti,kw OR (Pulmonary Inflammations):ab,ti,kw OR (Lung Inflammation):ab,ti,kw OR (Inflammation, Lung):ab,ti,kw OR (Inflammations, Lung):ab,ti,kw OR (Lung Inflammations):ab,ti,kw 7084  #10 #8 or #9 12561  #11 #10 and #7 14  #12 MeSH descriptor: [Mortality] explode all trees 14079  #13 MeSH descriptor: [Risk] explode all trees 39510  #14 (relative):ab,ti,kw OR (risk):ab,ti,kw OR (relative risk):ab,ti,kw OR (risks):ab,ti,kw OR (association):ab,ti,kw OR (Influencing factors):ab,ti,kw OR (risk factors):ab,ti,kw OR (risk factor):ab,ti,kw OR (Cause):ab,ti,kw OR (relevant factors):ab,ti,kw OR (mortality):ab,ti,kw 451975  #15 #12 or #13 or #14 456037  #16 #15 and #11 7 |
| **Web of Science** | #1 TS=(Hip Fractures OR Fractures, Hip OR Trochanteric Fractures OR Fractures, Trochanteric OR Fractures, Intertrochanteric OR Intertrochanteric Fractures OR Subtrochanteric Fractures OR Fractures, Subtrochanteric OR Femoral Neck Fractures OR Femoral Neck Fracture OR Femur Neck Fractures OR Femur Neck Fracture) 87227  #2 TS=(Pneumonia OR Pneumonias OR Lobar Pneumonia OR Lobar Pneumonias OR Pneumonia, Lobar OR Pneumonias, Lobar OR Experimental Lung Inflammation OR Experimental Lung Inflammations OR Inflammation, Experimental Lung OR Lung Inflammation, Experimental OR Lung Inflammations, Experimental OR Pneumonitis OR Pneumonitides OR Pulmonary Inflammation OR Inflammation, Pulmonary OR Inflammations, Pulmonary OR Pulmonary Inflammations OR Lung Inflammation OR Inflammation, Lung OR Inflammations, Lung OR Lung Inflammations) 491484  #3 #1 AND #2 933  #4 TS=(relative OR risk OR relative risk OR risks OR Influencing factors OR association OR risk factors OR risk factor OR Cause OR relevant factors OR mortality) 13974747  #5 #3 AND #4 764 |
| **Embase** | ('hip fracture'/exp OR 'fractures, hip':ab,ti OR 'trochanteric fractures':ab,ti OR 'intertrochanteric fractures':ab,ti OR 'fractures, trochanteric':ab,ti OR 'fractures, intertrochanteric':ab,ti OR 'subtrochanteric fractures':ab,ti OR 'fractures, subtrochanteric':ab,ti OR 'femoral neck fracture'/exp OR 'femoral neck fracture':ab,ti OR 'femur neck fractures':ab,ti OR 'femur neck fracture':ab,ti) AND ('pneumonia'/exp OR 'pneumonias':ab,ti OR 'lobar pneumonia':ab,ti OR 'lobar pneumonias':ab,ti OR 'pneumonias, lobar':ab,ti OR 'pneumonia, lobar':ab,ti OR 'experimental lung inflammation':ab,ti OR 'experimental lung inflammations':ab,ti OR 'inflammation, experimental lung':ab,ti OR 'lung inflammation, experimental':ab,ti OR 'lung inflammations, experimental':ab,ti OR 'pneumonitis':ab,ti OR 'pneumonitides':ab,ti OR 'pulmonary inflammation':ab,ti OR 'inflammation, pulmonary':ab,ti OR 'inflammations, pulmonary':ab,ti OR 'pulmonary inflammations':ab,ti OR 'lung inflammation':ab,ti OR 'inflammation, lung':ab,ti OR 'inflammations, lung':ab,ti OR 'lung inflammations':ab,ti) AND ('risk'/exp OR 'mortality'/exp OR 'relative':ab,ti OR 'risk':ab,ti OR 'relative risk':ab,ti OR 'risks':ab,ti OR 'association':ab,ti OR 'influencing factors':ab,ti OR 'risk factors':ab,ti OR 'risk factor':ab,ti OR 'cause':ab,ti OR 'relevant factors':ab,ti OR 'mortality':ab,ti) |
