# Supplementary material for: Risk factors for hospital-acquired pneumonia in hip fracture patients: a systematic review and meta-analysis
Source: BMC Musculoskelet Disord. 2024 Jan 2;25:6. doi: 10.1186/s12891-023-07123-0 (PMC10759764; doi:10.1186/s12891-023-07123-0)
Supplement: Supplementary file 3 — Additional file 3: PRISMA 2020 flow diagram for new systematic reviews which included searches of databases, registers and other sources. [file 12891_2023_7123_MOESM3_ESM.docx]

**Identification of studies via other methods**

**Identification of studies via databases and registers**

Records identified from:

Websites (n =0 )

Organisations (n =0 )

Citation searching (n = 0)

Records removed *before screening*:

Duplicate records removed (n = 1059)

Records identified from

Databases (n =2913 )

**Identification**

Records screened

(n = 1854)

Records excluded

(n = 1722)

Reports not retrieved(n = 88):

① Inappropriate study types (n=9)

② Inappropriate study participants (n=9)

③ Inappropriate study outcomes (n=35)

④ Inappropriate data (n=35)

Reports sought for retrieval

(n =132 )

**Screening**

Reports assessed for eligibility

(n =44 )

Reports excluded

(n =9 )

Studies included in review

(n =35 )

**Included**

*Consider, if feasible to do so, reporting the number of records identified from each database or register searched (rather than the total number across all databases/registers).

**If automation tools were used, indicate how many records were excluded by a human and how many were excluded by automation tools.

*From:*  Page MJ, McKenzie JE, Bossuyt PM, Boutron I, Hoffmann TC, Mulrow CD, et al. The PRISMA 2020 statement: an updated guideline for reporting systematic reviews. BMJ 2021;372:n71. doi: 10.1136/bmj.n71. For more information, visit: <http://www.prisma-statement.org/>
